# Genomic selection for growth characteristics in Korean red pine (*Pinus densiflora* Seibold & Zucc.)

**DOI:** 10.3389/fpls.2024.1285094

**Published:** 2024-01-23

**Authors:** Hye-In Kang, In Sik Kim, Donghwan Shim, Kyu-Suk Kang, Kyeong-Seong Cheon

**Affiliations:** ^1^ Division of Tree Improvement and Biotechnology, Department of Forest Bio-resources, National Institute of Forest Science, Suwon, Republic of Korea; ^2^ Department of Biological Sciences, Chungnam National University, Daejeon, Republic of Korea; ^3^ Department of Agriculture, Forestry and Bioresources, College of Agriculture and Life Sciences, Seoul National University, Seoul, Republic of Korea

**Keywords:** Korean red pine, progeny test, genomic selection, accelerated breeding, breeding value, genetic gain

## Abstract

Traditionally, selective breeding has been used to improve tree growth. However, traditional selection methods are time-consuming and limit annual genetic gain. Genomic selection (GS) offers an alternative to progeny testing by estimating the genotype-based breeding values of individuals based on genomic information using molecular markers. In the present study, we introduced GS to an open-pollinated breeding population of Korean red pine (*Pinus densiflora*), which is in high demand in South Korea, to shorten the breeding cycle. We compared the prediction accuracies of GS for growth characteristics (diameter at breast height [DBH], height, straightness, and volume) in Korean red pines under various conditions (marker set, model, and training set) and evaluated the selection efficiency of GS compared to traditional selection methods. Training the GS model to include individuals from various environments using genomic best linear unbiased prediction (GBLUP) and markers with a minor allele frequency larger than 0.05 was effective. The optimized model had an accuracy of 0.164–0.498 and a predictive ability of 0.018–0.441. The predictive ability of GBLUP against that of additive best linear unbiased prediction (ABLUP) was 0.86–5.10, and against the square root of heritability was 0.19–0.76, indicating that GS for Korean red pine was as efficient as in previous studies on forest trees. Moreover, the response to GS was higher than that to traditional selection regarding the annual genetic gain. Therefore, we conclude that the trained GS model is more effective than the traditional breeding methods for Korean red pines. We anticipate that the next generation of trees selected by GS will lay the foundation for the accelerated breeding of Korean red pine.

## 1 Introduction

Korean red pine (*Pinus densiflora* Siebold & Zucc.), a species native to South Korea, belongs to the genus *Pinus* of the family Pinaceae and is widely distributed throughout East Asia, from the Korean Peninsula to Japan and China (Szmidt and Wang, 1993). Considering its high timber value, the reforestation of Korean red pine accounted for approximately 17% of the annual reforestation area in South Korea as of 2020, highlighting the need for research on improving wood productivity by breeding economic traits (KFS, 2021).

Breeding is important for genetically improving trees by applying genetic principles and techniques (White et al., 2007). However, compared with crops and livestock, forest trees have a longer generation period, and the establishment of a breeding population by crossing and nurturing takes a long time. Consequently, the advancement of a generation requires 30–45 years for tree breeding. Currently, the most advanced tree-breeding program for loblolly pines (*Pinus taeda*) has progressed to the fourth generation (Isik and McKeand, 2019). As the response to selection, which is the expected value of improvement according to the progress of one generation, is limited, rapid generational advancement is required to achieve accelerated breeding.

Since the development of next-generation sequencing (NGS) and statistical analysis methods for large-scale data, genomic selection (GS) has been proposed as an alternative to traditional selection methods. GS is a selective breeding method that uses molecular marker information rather than phenotype or pedigree information to estimate the genetic value of each individual as a criterion for selection in the breeding population (Meuwissen et al., 2001). Unlike conventional family selection (FS) and marker-assisted selection, GS estimates breeding value by summing the effects of thousands to hundreds of thousands of markers, making it possible to discern individual distinctions caused by minute marker effects (Goddard et al., 2011). Additionally, GS focuses solely on selection efficiency and does not require any prior information, such as the association between the phenotype and marker, location of the quantitative trait loci on the genome, or the relative influence of the marker on the phenotype (Isik et al., 2016). Accordingly, the time and effort required to find information on the association between a specific marker and the trait can be saved in GS. In addition, GS is particularly advantageous for forest trees because GS does not reply on reference genomes.

Studies on GS in loblolly pine and eucalyptus hybrids marked the first application of this approach to forestry (Resende et al., 2012; Resende et al., 2012a; Resende et al., 2012b). Subsequently, experimental studies on GS in conifers such as *Pinus*, *Picea*, *Pseudotsuga*, and *Cryptomeria* and broadleaf trees such as *Eucalyptus*, *Castanea*, *Fraxinus*, and *Populus* have been conducted (reviewed in Lebedev et al., 2020). Previous studies on forest trees employed population sizes of 25–338 half-sibling or full-sibling families as research subjects, primarily focusing on assessing their growth and wood quality attributes.

Generally, the GS in forest trees proceeds as follows: First, the genotypes and phenotypes of the training group in the breeding population are evaluated. Second, the two datasets are combined to create a prediction model that simultaneously estimate the effects of all marker loci. Third, cross-validation is performed to test the applicability of the developed model. Finally, genetic values are predicted for different subgroups of the breeding population, and individuals for advanced generations are selected based on the estimated genomic values (Grattapaglia, 2014).

In this study, we proposed an efficient GS model for Korean red pine by investigating the impact of various factors, including the single nucleotide polymorphism (SNP) marker set, predictive model, and training dataset, on prediction accuracy in the half-sib population. Additionally, the prediction accuracy of the trained GS model was evaluated through standardization. Moreover, the efficiency of GS was evaluated by comparing the response to GS and conventional selections.

## 2 Materials and methods

### 2.1 Study population

The study population was from an open-pollinated progeny trial established for the progeny test of plus trees by the National Institute of Forest Sciences (NIFoS). Seeds obtained from 49 grafted clones (20 and 29 clones of plus trees from Gangwon and Kyeongbuk provinces, respectively) of the clone bank located in Taean were nursed to produce 1-1 seedlings. Subsequently, the seedlings were distributed and planted in 6 regions: Taean (36°46’ N, 126°38’ W), Chuncheon (37°89’ N, 127°63’ W), Gongju (36°60’ N, 127°10’ W), Kyeongju (35°92’ N, 129°09’ W), Naju (35°06’ N, 126°84’ W), and Wanju (35°85’ N, 127°28’ W). A total of 2,643 trees for which both phenotypes and genotypes were investigated from the open-pollinated progeny trial were used for the analysis: 609 from Taean, 726 from Chuncheon, 456 from Gongju, 380 from Kyeonju, 247 from Naju, and 225 from Wanju. These trees belonged to 44 open-pollination families (**Supplementary Table 1**).

### 2.2 Phenotyping

The target traits of GS were growth, including diameter at breast height (DBH), height, straightness, and volume. Laser scanning technology, which is capable of consistently generating high-precision outputs as well as environmentally friendly because of its nondestructive properties (Dittmann et al., 2017; Chen et al., 2019), was used to replace the conventional survey. To obtain the phenotype of each individual, point cloud data from six sites in the study population were obtained using a LiDAR device (ScanStation P40, Leica) in 2017 and 2018. For batch data processing, the point-cloud data were separated into trees and ground, and the ground was flattened. The DBH (m) was obtained by dividing the circumference of the tree at a height of 1.2 m from the ground by the circumference ratio (π). Tree height (m) was measured as the distance from the ground to the top of the canopy. The volume (m^3^) of each tree was calculated as (DBH)^2^ × (height). To calculate straightness, an imaginary baseline was written connecting the center point of the tree trunk at the root collar and a height of 6 m. The distances from the baseline to the center point of the tree at heights of 0.5, 1.5, 2.5, 3.5, 4.5, and 5.5 m were calculated. The straightness was digitized by taking the negative natural log (-log_e_) of the standard deviation of the six distance values. Specifically, the more the stem had a shape similar to the baseline, the greater the calculated straightness. The distribution of the phenotypic data used in this study is shown in **Supplementary Figure 2.**.

### 2.3 Genotyping

To investigate the genotype of each tree at the six test sites, cambium tissues were collected from tree stems, and DNA was extracted using the Exgene™ Plant SV Kit (Geneall, Seoul). SNP probe information for genotyping was obtained using a 50 K SNP chip for Korean red pines (Cheon et al., 2021). The SNP chip was developed using genotyping-by-sequencing (GBS) information from 46 trees tested in the open-pollinated progeny trial. Axiom™ analysis suite (v5.0.1) was used for SNP calling. First, the sample quality was investigated, and samples with a sample call rate of less than 97% or a DQC value of less than 0.82 based on probe intensity were excluded. The genotypes (AA, BB, AB, and NN) were clustered for each SNP marker based on the intensity ratio of the two allele probes.

### 2.4 Preparation of GRM

As there should be no missing genotype data to write a genomic realized relationship matrix (GRM), imputation was performed using Beagle (v5.2), which operates even without a reference genome (Browning and Browning, 2009). GRMs were prepared according to the formula described by VanRaden (2008). For comparison with the GRM, a numerator relationship matrix (NRM) consisting of the relationship coefficients of two individuals was also written using pedigree information. The GRMs differed according to the marker set used, but the coefficients of the GRM and NRM commonly showed approximate matches (**Supplementary Figure 3**).

### 2.5 Heritability

The heritability of the mixed model was obtained as the variance of NRM, and the GRM was divided by the total variance in the best linear unbiased prediction (BLUP).


$$h_{NRM}^{2}=\;\frac{\sigma_{NRM}^{2}}{\sigma_{NRM}^{2}+\;\sigma_{r}^{2}},\;h_{GRM}^{2}=\;\frac{\sigma_{GRM}^{2}}{\sigma_{GRM}^{2}+\;\sigma_{r}^{2}}$$

The site and block were set as the fixed effects of the mixed linear model in the combined and one-site analyses, respectively.

Family heritability was estimated using analysis of variance (ANOVA). The mathematical linear model for analyzing the variance component and heritability of each site was as follows:


$$X_{ijk}=\;\mu +B_{i}+\;F_{j}+\;BF_{ij}+\;\epsilon_{ijk}$$

where X_ijk_ is the phenotype of the k-th tree of the j-th family in the i-th block. μ is the overall mean, B_i_ is the effect of the block, F_j_ is the effect of family, BF_ij_ is the interaction effect of block and family, and ϵ_ijk_ is the error term. The mathematical linear model for the combined analysis of the six sites is as follows:


$$X_{ijkl}=\;\mu +S_{i}+B_{ij}+\;F_{k}+\;SF_{ik}+BF_{ijk}+\;\epsilon_{ijkl}$$

where X_ijkl_ is the phenotype of the l-th tree of the k-th family in the j-th block of the i-th site, μ is the overall mean, S_i_ is the effect of the site, B_ij_ is the effect of the block, F_k_ is the effect of the family, SF_ik_ is the interaction effect of the site and family, BF_ij_ is the interaction effect of the block and family, and ϵ_ijk_ is the error term.

### 2.6 SNP marker selection

To compare the prediction accuracy according to the SNP calling the quality of markers, markers selected based on loose, moderate, and strict standards were used for GS. The loose standard was the use of all markers, the moderate standard was to meet the default quality threshold of the SNP calling program (call rate (CR)≥97% and Fisher’s linear discriminant (FLD)≥3.6), and the strict standard was the addition of the threshold of CR≥99%, FLD≥5, heterozygous strength offset (HetSO)≥0, and homozygote ratio offset (HomRO)≥0. In common, markers with minor allele frequency (MAF) of 0.05 or higher were used.

To study the prediction accuracy according to the number of markers, all 17 K (17,074) markers showing genotype variation and 2 K (2,000), 6 K (6,000), and 10 K (10,000) markers randomly selected markers were used for GS. In addition, markers with MAF of 0.25, 0.05, and 0.0005 or higher were selected and compared to investigate the impact of MAF on prediction accuracy.

### 2.7 Genomic estimated breeding value prediction

Six genomic predictive models, including genomic BLUP (GBLUP), Bayesian least absolute shrinkage and selection operator (LASSO), Bayesian Ridge Regression, Bayes A, Bayes B, and Bayes C, were used to predict genomic estimated breeding value (GEBV). In addition, additive BLUP (ABLUP), a pedigree-based prediction method using NRM, was compared. BLUPs were performed using remlf90 in the R package BreedR (v0.12.5). In addition, five Bayesian models were applied with 20,000 iterations using the GBLR function of the R package BGLR (v1.0.8) (Pérez and de Los Campos, 2014).

### 2.8 Genomic selection scenario and evaluating prediction accuracy

To evaluate the prediction accuracy of GS, the data set was split into the training set, of which both genotype and phenotype were used for training the model, and the test set, of which phenotype were masked, so to be predicted by model. In this study, three genomic selection scenarios were performed according to how training set and test set were defined: within-region prediction, between-region prediction, and combined-region prediction. Three-, five-, ten-, and twenty-fold cross-validations were examined and compared in a within-region prediction scenario. In the between-region prediction scenario, all individuals in the remaining five regions (training set) were trained to predict the GEBV for one region (test set). In addition, in the combined-region prediction scenario, 10-fold cross-validation was performed by randomly dividing the groups regardless of the region. When multiple regions were included in a scenario, the phenotype was corrected by setting the region as a fixed effect in the linear model.

The prediction accuracy was evaluated using accuracy (AC) and predictive ability (PA). Accuracy was the Pearson correlation coefficient of the GEBV of the test set and the EBV calculated by ABLUP using pedigree information and all phenotypes (**Supplementary Figure 4**), which we assume as true breeding value (TBV) as in traditional genetics and majority of forest tree GS studies (Chen et al., 2018; Li et al., 2019; Beaulieu et al., 2020). The predictive ability was the Pearson correlation coefficient between the GEBV of the test set and the phenotype. In cross-validation, prediction accuracy is the average values of correlation coefficients for each subset. For a meaningful comparison, we also presented the average correlation coefficient for the same subset in between-region prediction.

### 2.9 Response to selection

To compare the response to the selection of GS and two traditional selections, FS and phenotypic selection (PS), the annual genetic gain of each was calculated. Genetic gains from FS and PS were calculated as follows (Voss-Fels et al., 2019).


$$\Delta G_{P}=\frac{ih^{2}\sigma_{P}}{t}$$

where ΔG_P_ is the annual genetic gain, i is the selection intensity, h^2^ is h_GRM_^2^ for PS and family heritability by ANOVA (**Supplementary Table 5**) for FS, σ_P_ is the square root of phenotypic variance for PS and family phenotypic variation for FS, and t is breeding cycle. The genetic gain from GS was measured as follows (Isik et al., 2017; Voss-Fels et al., 2019).


$$\Delta G_{A}=\frac{ir\sigma_{A}}{t}$$

where ΔG_A_ is the annual genetic gain, i is the selection intensity, r is the accuracy of GS, σ_A_ is the square root of the additive genetic variance, and t is the breeding cycle. The selection intensity, according to the selection ratio, was calculated assuming a normal phenotypic distribution.

The breeding cycle of the GS was assumed to be 15 years, considering the age of reproduction of Korean red pine. For the FS, the time required for the progeny test (30 years in the study population) was added. Accordingly, a total breeding cycle of 45 years was assumed for the FS. In addition, for PS, the breeding cycle was assumed to be 30 years because of the time required to grow to the phenotyping age.

## 3 Results

### 3.1 Heritability

To estimate narrow-sense individual heritability and breeding values in this study, a mixed model was employed instead of the traditionally used ANOVA as the latter is not suitable for unbalanced data with different observation numbers per family and block (Isik et al., 2017). The relationship matrices, GRM and NRM, were used as random effects of the mixed model. The heritability of the population ranged from 0.000 to 0.723, exhibiting substantial variability across test sites and traits (**Table 1**). Height displayed the highest heritability among all the assessed traits. NRM and GRM heritability estimates showed a similar trend depending on the test sites and traits. Notably, the heritability values derived from the GRM were generally higher than those derived from the NRM.

**Table 1:** Narrow-sense heritability by the mixed model using NRM and GRM in progeny test sites.

Region ^a^	Estimates	DBH	Height	Straightness	Volume
T	*h_NRM_^2^*	0.103 (0.083)	0.249 (0.124)^*^	0.078 (0.092)	0.143 (0.092)
	*h_GRM_^2^*	0.186 (0.106)	0.723 (0.103)^**^	0.146 (0.105)	0.276 (0.112)^*^
C	*h_NRM_^2^*	0.195 (0.101)	0.380 (0.148)^*^	0.045 (0.060)	0.208 (0.103)^*^
	*h_GRM_^2^*	0.420 (0.099)^**^	0.393 (0.098)^**^	0.227 (0.095)^*^	0.463 (0.105)^**^
G	*h_NRM_^2^*	0.407 (0.187)^*^	0.457 (0.200)^*^	0.245 (0.144)	0.413 (0.190)^*^
	*h_GRM_^2^*	0.354 (0.151)^*^	0.720 (0.138)^**^	0.356 (0.146)^*^	0.393 (0.150)^**^
K	*h_NRM_^2^*	0.055 (0.115)	0.000 (0.000)	0.327 (0.193)	0.001 (0.003)
	*h_GRM_^2^*	0.051 (0.105)	0.564 (0.197)^**^	0.405 (0.171)^*^	0.004 (0.091)
N	*h_NRM_^2^*	0.068 (0.155)	0.132 (0.179)	0.366 (0.277)	0.043 (0.151)
	*h_GRM_^2^*	0.546 (0.228)^*^	0.642 (0.215)^**^	0.395 (0.291)	0.519 (0.225)^*^
W	*h_NRM_^2^*	0.070 (0.188)	0.108 (0.212)	0.000 (0.003)	0.206 (0.235)
	*h_GRM_^2^*	0.173 (0.175)	0.105 (0.187)	0.286 (0.245)	0.173 (0.187)
Combined	*h_NRM_^2^*	0.074 (0.031)^*^	0.146 (0.049)^**^	0.113 (0.042)^**^	0.108 (0.040)^**^
	*h_GRM_^2^*	0.066 (0.017)^**^	0.104 (0.020)^**^	0.059 (0.017)^**^	0.082 (0.018)^**^

Standard error of heritability estimation in parenthesis.

The values marked with asterisk indicate a difference from zero (*, α<0.05; **, α<0.01).

a T, Taean; C, Chuncheon; G, Gongju; K, Kyeongju; N, Naju; W, Wanju.

### 3.2 Impact of SNP marker set on predictive accuracy

To determine the impact of SNP marker quality on the prediction accuracy of GS, GBLUP analysis was performed using different marker quality standards, and the resulting accuracies and predictive abilities were compared. The number of markers meeting the standards was 6,464 for the loose standard, 1,164 for the moderate standard, and 571 for the strict standard, respectively. Across all the traits and regions, the stricter the applied standard, the lower the accuracy and predictive ability (**Supplementary Table 6**). Although some data showed differences within the error range, all traits in Chuncheon exhibited significant differences in accuracy across the different marker sets. In the subsequent analysis, a loose standard was applied for the selection of markers to achieve relatively high GS accuracy.

**Figure 1:**
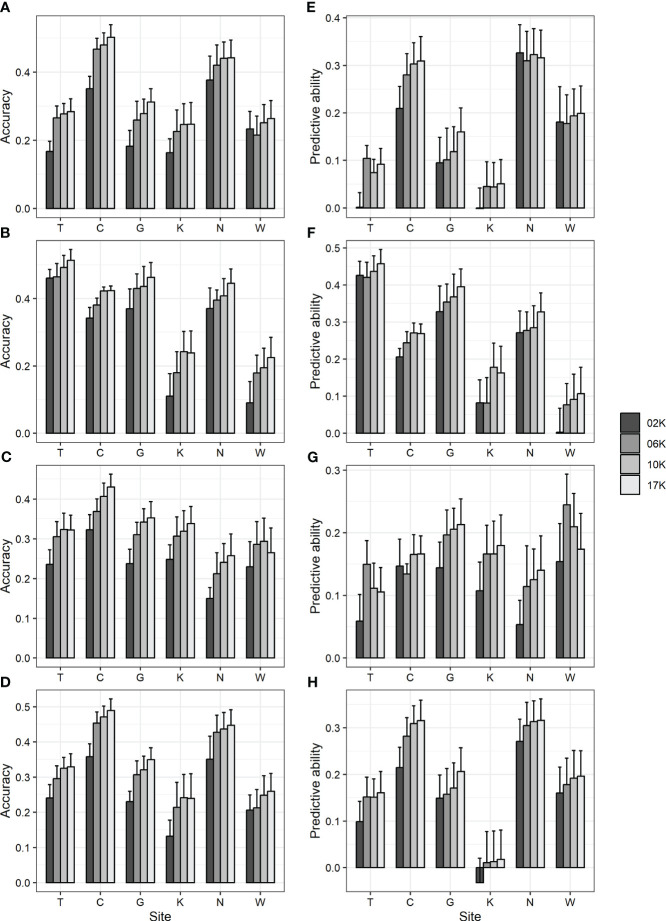
GBLUP accuracy and predictive ability using 17 K (17,074) markers showing genotype variation and 2 K (2,000), 6 K (6,000), and 10 K (10,000) markers randomly selected from them. **(A, E)** DBH **(B, F)** height **(C, G)** straightness and **(D, H)** volume. **(A-D)** accuracy **(E-H)** predictive ability. Bar and error bar are mean and standard error of accuracy and predictive ability from 10-fold cross-validation.

To examine the impact of the number of markers, all 17 K (17,074) markers showing genotype variation and 2 K (2,000), 6 K (6,000), and 10 K (10,000) marker sets randomly selected from among them were used in GBLUP, and prediction accuracy was compared. As a result, the accuracy was 0.09–0.46 for 2 K, 0.18–0.47 for 6 K, 0.24–0.48 for 10 K, and 0.22–0.51 for 17 K marker sets (**Figures 1A-D**; **Supplementary Table 7**). Also, the predictive ability was -0.03–0.43 for 2 K, 0.01–0.42 for 6 K, 0.01–0.44 for 10 K, and 0.02–0.46 for 17 K marker sets (**Figures 1E-H**; **Supplementary Table 7**). Generally, as the number of markers decreased, the prediction accuracy decreased.

Subsequently, an analysis was conducted to investigate whether a reduction in the number of MAF markers would result in a decrease in prediction accuracy. The prediction accuracy was compared using GBLUP analysis with 2 K (2,248), 6 K (6,464), and 10 K (9,799) markers with MAF of 0.25, 0.05, and 0.0005 or higher, respectively. As a result, the accuracy was 0.14–0.5, and the predictive ability was -0.02–0.46, showing differences within the error range (**Supplementary Table 8**). Specifically, when markers with low MAF were excluded, the prediction accuracy did not decrease, unlike when random markers were excluded.

### 3.3 Impact of the predictive model on predictive accuracy

**Figure 2:**
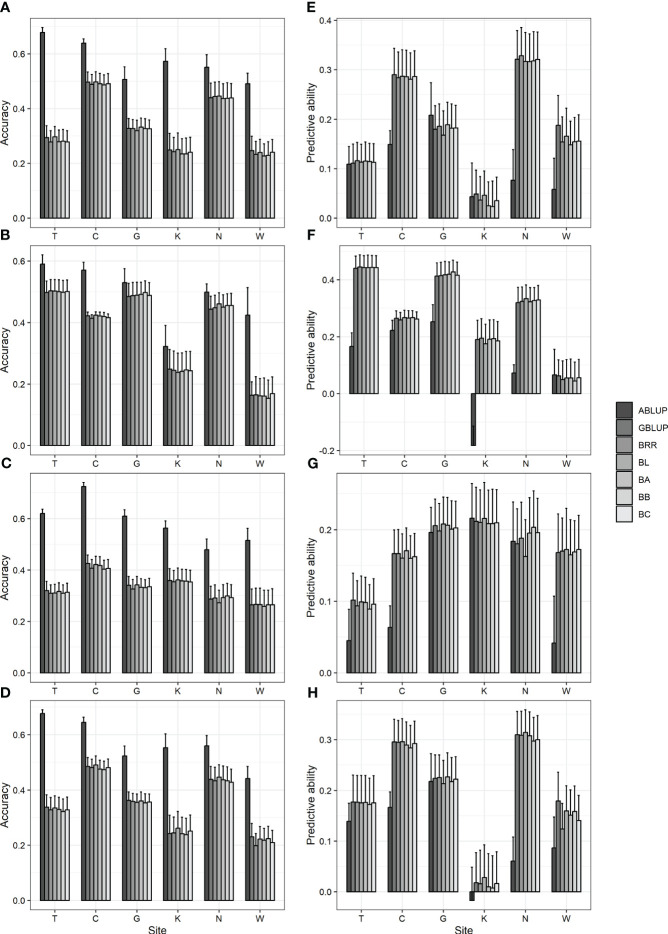
Accuracy and predictive ability by ABLUP and genomic selection models, including GBLUP and five Bayesian models. **(A, E)** DBH **(B, F)** height **(C, G)** straightness and **(D, H)** volume. **(A-D)** accuracy **(E-H)** predictive ability. BRR, Bayesian ridge regression; BL, Bayesian LASSO; BA, Bayes A; BB, Bayes B; BC, Bayes C. Bar and error bar are mean and standard error of accuracy and predictive ability from 10-fold cross-validation.

The prediction accuracies of various models were analyzed and compared to identify a suitable predictive model for GS in Korean red pine. In addition, ABLUP was performed to compare the efficiency of GS with pedigree-based selection. As a result, six GS models exhibited an accuracy of 0.15–0.5 and a predictive ability of 0.01–0.44, with no significant differences observed among one another (**Figure 2**; **Supplementary Table 9**). Meanwhile, compared with ABLUP (accuracy 0.32–0.72, predictive ability -0.18–0.25) based on pedigree information, the GS models generally showed lower accuracy and higher predictive ability (**Figure 2**; **Supplementary Table 9**), which may likely be attributed to the fact that the phenotype is not strongly correlated with the initially assumed TBV (**Supplementary Figure 4**).

### 3.4 Impact of training data set on predictive accuracy

To examine whether the size of the training and test sets affected the prediction accuracy of the GS of Korean red pine, the accuracy and predictive ability of GBLUP were compared by varying the number of cross-validation folds. As a result of 3-, 5-, 10-, and 20-fold cross-validations, the accuracy was 0.14–0.51, and the predictive ability was -0.07–0.44, showing differences within the error range regardless of the size or ratio of the training set (**Supplementary Table 10**). For some regions and traits, such as the volume in Naju, the prediction accuracy increased as the training set size increased.

**Figure 3:**
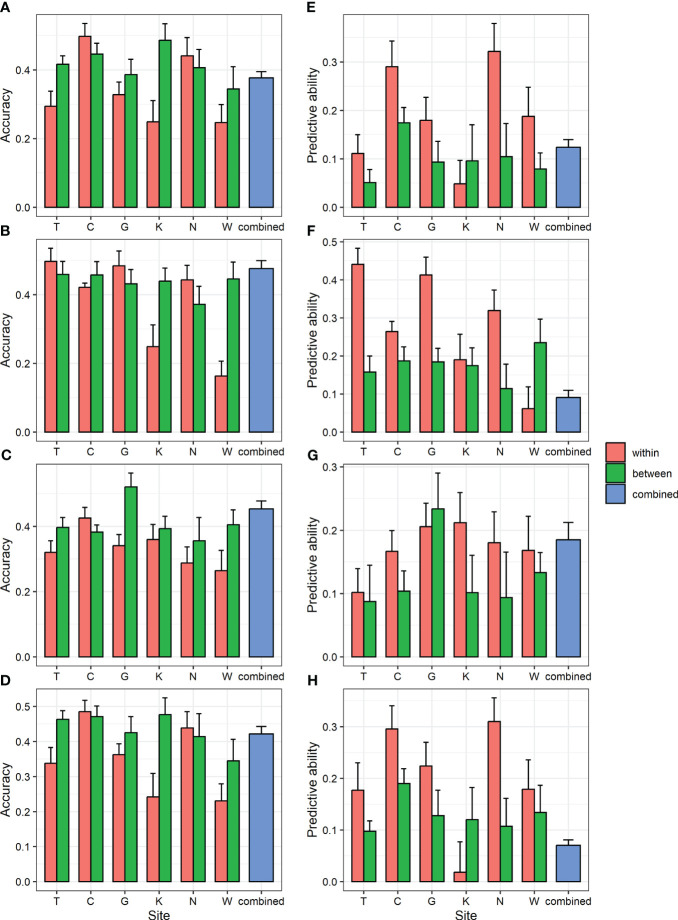
GBLUP accuracy and predictive ability of within- and between-region analysis and combined region analysis for four traits. **(A, E)** DBH **(B, F)** height **(C, G)** straightness and **(D, H)** volume. **(A-D)** accuracy **(E-H)** predictive ability. Bar and error bar are mean and standard error of accuracy and predictive ability from 10-fold cross-validation.

To determine whether GS can be applied to populations in different environments, within-, between-, and combined-region GS scenarios were compared (**Figure 3**; **Supplementary Table 11**). Accuracy did not show consistent results depending on whether the analysis was within or between regions. However, the predictive ability was generally higher in the within-region prediction (0.02–0.41) than in the between-region prediction (0.05–0.24). In the combined-region scenario, the accuracy was 0.38–0.48, which was higher than the average of six regions (0.33–0.38), and the predictive ability was 0.07–0.18, which was lower than the average of the six regions (0.17–0.28). Higher accuracy could be obtained when the GS model was trained by combining multiple environments, consistent with previous study in two spruce species (Lenz et al., 2017; Chen et al., 2018).

### 3.5 Prediction accuracy evaluation

Synthesizing the study results, the optimized GS model for Korean red pine was the GBLUP model, using the genotypes of 6,464 markers with an MAF of 0.05 or higher. Finally, the prediction accuracy of the GS performed by this model was 0.164–0.498 for accuracy and 0.018–0.441 for predictive ability (**Table 2**). The prediction accuracy was highest for height in Taean. The mean prediction accuracy for all regions was the highest for height. The population size and prediction accuracy were not correlated.

**Table 2:** Prediction accuracy for four traits using GBLUP of 10-fold cross-validation.

Trait	Prediction accuracy ^a^	Region ^b^							
		T	C	G	K *	N	W	Mean	Combined
DBH	AC	0.294	0.497	0.328	0.249	0.441	0.267	0.346	0.377
	PA	0.111	0.29	0.18	0.049	0.322	0.188	0.19	0.124
	G/A	1.022	1.948	0.864	1.117	4.197	3.23	–	1.223
	G/H	0.257	0.447	0.303	0.217	0.436	0.452	–	0.483
Height	AC	0.498	0.422	0.485	0.249	0.443	0.164	0.377	0.476
	PA	0.441	0.264	0.413	0.19	0.32	0.062	0.282	0.091
	G/A	2.664	1.189	1.633	–	4.41	0.94	–	1.159
	G/H	0.519	0.421	0.487	0.253	0.399	0.191	–	0.282
Straight- ness	AC	0.32	0.426	0.341	0.36	0.288	0.265	0.333	0.453
	PA	0.102	0.167	0.206	0.212	0.18	0.168	0.173	0.185
	G/A	2.258	2.626	1.048	0.981	0.981	4.038	–	1.558
	G/H	0.267	0.351	0.345	0.333	0.286	0.314	–	0.762
Volume	AC	0.338	0.485	0.363	0.242	0.439	0.231	0.35	0.421
	PA	0.177	0.296	0.224	0.018	0.31	0.179	0.201	0.07
	G/A	1.274	1.775	1.029	–	5.098	2.067	–	0.539
	G/H	0.337	0.435	0.357	0.285	0.43	0.43	–	0.244

a AC, accuracy, r(GEBV, EBV_im_), EBV_im_ is estimated using ABLUP with all phenotypic data; PA, predictive ability, r(GEBV, phenotype); G/A, Predictive ability of GBLUP against that of ABLUP; G/H, Predictive ability of GBLUP against the square root of heritability.

b T, Taean; C, Chuncheon; G, Gongju; K, Kyeongju; N, Naju; W, Wanju.

^*^ Values for height and volume were omitted because the predictive ability of ABLUP was negative.

**Figure 4:**
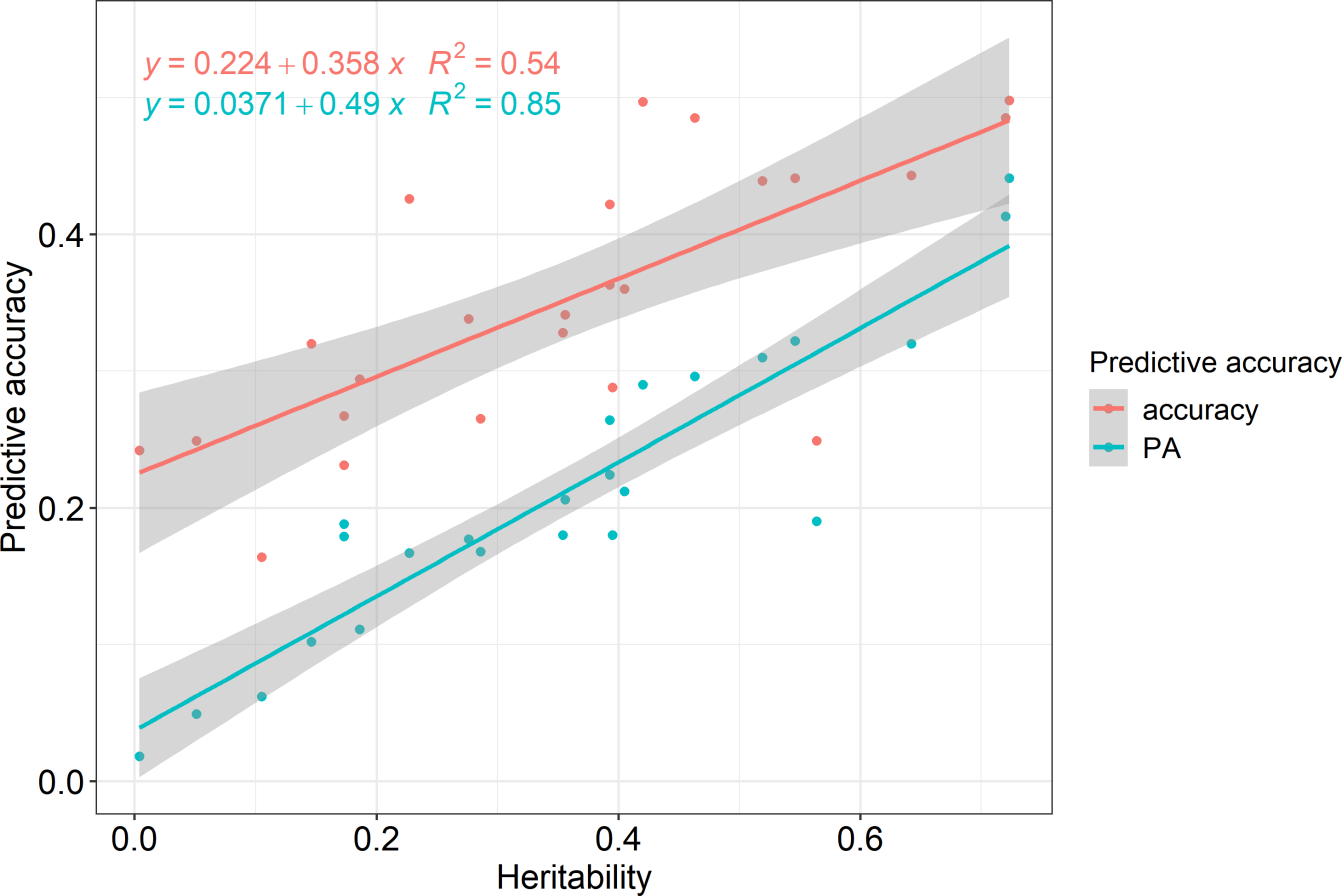
Prediction accuracies according to heritability.

Pearson correlation analysis was performed for each region and trait to reveal the correlation between heritability and prediction accuracy of GS in Korean red pine. The correlation coefficient between accuracy and heritability was 0.735 (p<0.001), and that between predictive ability and heritability was 0.924 (p<0.001), indicating a strong correlation (**Figure 4**).

### 3.6 Response to selection

**Figure 5:**
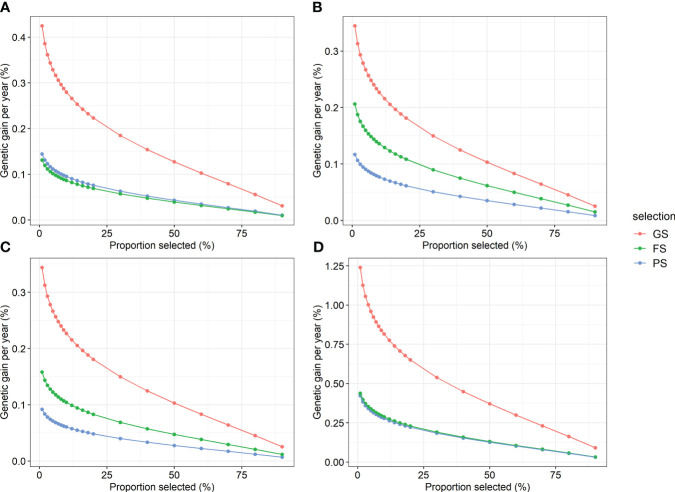
Annual genetic gain of genomic selection and two traditional selections by proportion selected for four traits. **(A)** DBH, **(B)** height, **(C)** straightness, and **(D)** volume. GS, genomic selection; FS, family selection; PS, phenotypic selection.

To test the efficiency of GS versus traditional breeding, the genetic gains expected to be obtained using GS, PS, and FS were compared. Regarding the annual genetic gain in the combined region analysis, GS was the highest, followed by FS, and PS was the lowest for height, straightness, and volume (**Figure 5**). The efficiency of GS was superior to that of the two traditional selection methods for DBH. GS showed an annual genetic gain equivalent of 2.9–3.7 times PS and 1.7–3.2 times FS when 20% selection was conducted. In addition, the annual genetic gain of GS was the highest at 0.09–1.24% volume. In addition, GS was the most efficient of the three selection methods in the within-region analysis (**Supplementary Table 12**). In the GS within the region, a response to selection of up to 1.9% per year was obtained.

## 4 Discussion

Breeding woody plants is a time- and cost-intensive process compared with that of crops, primarily because trees have a lengthy juvenile period, take longer to flower and produce seeds, and are physically larger than crops. Progeny tests for forest trees require a vast area and prolonged observation. In addition, as forest tree utilization continues to diversify and global climate change intensifies, the target traits of tree breeding are rapidly changing. Therefore, accelerated breeding is crucial for forestry.

The major advantage of GS in forest trees is that the selection efficiency can be improved by reducing the generation interval through genomic information-based selection before the phenotypes are expressed (Grattapaglia and Resende, 2011). Moreover, GS can increase selection intensity, resulting in a greater response to selection (Isik, 2014; Grattapaglia, 2017). Therefore, if GS is introduced into the breeding of Korean red pine, we can expect significant improvements by accelerating generations at a relatively low cost through early and intense marker-based selection rather than relying solely on progeny tests.

### 4.1 Heritability predictive accuracy

Heritability has been reported to have a significant effect on the prediction accuracy of GS. The prediction accuracy of GS increased as heritability increased in loblolly pine (Resende et al., 2012a), and the square root of heritability was strongly correlated with GS accuracy (Lian et al., 2014). However, in large training populations of 1,000 or more, heritability has a relatively small effect on GS accuracy compared to other factors (Grattapaglia and Resende, 2011).

Heritability estimates obtained using the GRM in this study were generally higher than those obtained using the NRM for each region (**Table 1**). This difference is because the relationship coefficients of the NRM may not accurately reflect the actual relationships among individuals. In the present study, the kinship coefficient of open-pollinated family progenies was assumed to be 0.25, based on the half-sib family assumption (Wright, 1922). However, in the real world, kinship relationships among open-pollinated siblings might be stronger because of various factors, including self-pollination, self-half, half-sib, full-sib, and common ancestry between female and male parents (Askew and El-Kassaby, 1994).

According to the correlation analysis, the prediction accuracy of GS in the Korean red pine was strongly influenced by heritability (**Figure 4**). Predictive ability showed a stronger correlation with heritability than with accuracy because it was calculated by phenotype, which implied the effects of environment and non-additive genetic variance. Therefore, heritability must be considered when performing GS on Korean red pines.

### 4.2 Model optimization

The essential stage of GS is training the model to estimate the effects of all markers. This stage includes optimizing the model to achieve the best prediction efficiency. We investigated the impact of the marker set, predictive model, and training dataset on the prediction accuracy of the model optimization for GS in Korean red pine.

Ensuring the quality of the SNP array data is crucial because it significantly affects the accuracy and precision of subsequent analyses. Contaminated data may lead to false-positive or false-negative results, underscoring the importance of controlling the data quality (Yang et al., 2011). Although previous studies on GS in forest trees have generally set a call rate criterion of 85–95% (Beaulieu et al., 2014; Cappa et al., 2019; Ukrainetz and Mansfield, 2020a), the impact of marker quality on the accuracy of GS has not been extensively explored. This study compared the effects of marker set size and quality on GS accuracy in Korean red pines (**Supplementary Table 6**). Notably, we found that including markers of slightly lower quality but increasing the number of markers was more effective in improving the accuracy of GS than focusing only on high-quality markers. This may be because the imputation of missing genotypes could compensate for the low call rates of the markers. Previous studies have shown that adding markers with low call rates can improve prediction accuracy when the markers are not saturated in the whole genome (Rutkoski et al., 2013). This suggests that the 500–1000 SNP markers in the array used in this study may not be sufficient to capture the entire Korean red pine genome. Another possible explanation for the higher accuracy of the imputed data is that imputation can capture associations among closely related individuals that might have been missed due to missing data (Weigel et al., 2010; Rutkoski et al., 2013).

The number of markers used in GS is a critical factor that affects the prediction accuracy as well as the computational time required for the analysis. Therefore, identifying the optimal number of markers for GS analysis is important. Our study showed that the MAF had a more significant impact on the prediction accuracy of GS than the number of markers (**Supplementary Tables 7**, **8**). Specifically, for height in Chuncheon and Kyeongju, we observed a significant decline in the prediction accuracy and predictive ability when the MAF was below 0.25 (2 K), suggesting that useful markers for prediction were present between the MAF range of 0.05 and 0.25. This MAF range was consistent with the selection of markers based on an MAF of 0.005–0.05 in previous studies on the GS of forest trees (Beaulieu et al., 2014; Cappa et al., 2019; Ukrainetz and Mansfield, 2020a). Therefore, our study supports the conclusion that selecting markers based on an MAF of 0.05 is efficient for GS analysis of Korean red pine, consistent with previous studies.

The GBLUP model was originally proposed as a predictive model for GS, and subsequent developments have led to the application of Bayesian models in various situations. However, most studies on the quantitative traits of forest trees, including spruce hybrids, blue gum (*Eucalyptus globulus*), maritime pine (*Pinus pinaster*), lodgepole pine (*Pinus contorta*), and Japanese cedar (*Cryptomeria japonica*), have not found any significant advantage in a particular model (Ratcliffe et al., 2015; Isik et al., 2016; Durán et al., 2017; Hiraoka et al., 2018; Ukrainetz and Mansfield, 2020b). Factors such as overfitting the training population and computing time should be considered when the accuracies of all models are similar (Heslot et al., 2012). Considering the similar accuracy and predictive ability of the six models tested in this study and that GBLUP was nine times faster than the Bayesian methods, we concluded that GBLUP is a more efficient choice for the GS of Korean red pine.

Generally, the ratio of training set size to test set size did not affect predictive accuracy (**Supplementary Table 10**), which was consistent with the results for Norway spruce (*Picea abies*) (Chen et al., 2018) and eucalyptus (Tan et al., 2017). This suggests that other factors, such as population structure and environment, were more important than the ratio of the training set when the population size was 200–700 for the GS of Korean red pine.

### 4.3 Environment effect on GS

The interaction between genotype and environment (G×E) refers to the inconsistency in the expression of traits when individuals are grown in different environments. Typically, there is greater interaction when clones or families change ranks across different environments. Because GS ranks individuals according to the GEBV, considering G×E when developing GS strategies is important (Grattapaglia, 2017). The predictive ability was lower in the between-region scenario than that in the within-region scenario (**Figure 3**; **Supplementary Table 11**), indicating a strong G×E in study population. This result was further supported by the generally low type-B genetic correlation ( $r_{12}=\;\sigma_{a12}/\sqrt{\sigma_{a1}^{2}\sigma_{a2}^{2}\;}\;$ ) and changing phenotype ranks of family across six sites (**Supplementary Table 13**, **Supplementary Figure 14**). Training across a range of environments may be beneficial to increase the probability of including similar environments for new populations in the training dataset. As a direction for further research, an approach worth considering is the utilization of models that take environmental factors into account, as exemplified in some crop studies (Burgueño et al., 2012; Jarquín et al., 2014; Sukumaran et al., 2017).

### 4.4 The efficiency of GS in Korean red pine

Similar to many previous studies on black spruce (*Picea mariana*), white spruce (*Picea glauca*), and lodgepole pine (Lenz et al., 2017; Beaulieu et al., 2020; Lenz et al., 2020a; Ukrainetz and Mansfield, 2020b), the predictive ability of GS models was higher than those of FS and PS in Korean red pine, confirming that GS for obtaining GEBV using DNA markers has an advantage in phenotype prediction over traditional selection in forest trees.

The prediction accuracy of GS is influenced by population-specific features, such as heritability, making direct comparisons across different populations unreliable. Instead, standardization using measures, such as the prediction accuracy of ABLUP or heritability, can be used for more accurate comparisons. Within-region analysis showed that the predictive ability of GBLUP compared to ABLUP ranged from 0.864 to 5.098 (**Table 2**), which was higher than the range of 0.80 to 0.95 reported for Norway spruce (Chen et al., 2018). The predictive ability of GBLUP against the square root of heritability was 0.19 to 0.76 (**Table 2**), which was lower on average than the result for Norway spruce (higher than 0.69) but similar to the result for white spruce (Beaulieu et al., 2020; Lenz et al., 2020b). Therefore, GS in Korean red pine was as efficient as in previous forest tree studies.

The annual genetic gain comparison revealed that GS was more efficient than the conventional selection methods (**Figure 5**). Moreover, selection intensity in the GS was enhanced. Because selection can be conducted at the seedling stage in GS, whereas PS and FS are conducted at the age after phenotype expression, the selection intensity could be increased in GS for the same number of selected trees (Grattapaglia, 2017). Therefore, in terms of annual genetic gain, the GS of Korean red pine was judged to be more efficient than the two traditional selection methods in both within- and combined-region scenarios.

### 4.5 Prospective of GS of Korean red pine

Statistical GS models such as GBLUP and Bayesian models are constrained by various factors, such as their underlying assumption of additive effects, limited capacity to capture non-linear relationships, and the difficulty they encounter when handling extensive datasets (Budhlakoti et al., 2022). Conversely, machine learning-based GS methods provide a more versatile and scalable approach, adept at capturing intricate non-linear relationships, rendering them particularly well-suited for addressing various genetic scenarios (Montesinos-López et al., 2021a; Montesinos-López et al., 2021b). Given the substantial impact of environmental factors and the prevalence of missing genotype data in this study, exploring deep learning-based genomic selection is essential for future research endeavors in GS of Korean red pine.

## 5 Conclusion

In this study, an efficient GS model for Korean red pine was presented, and its selection efficiency was evaluated. As a result of comparing various marker subsets, predictive models, and scenarios to optimize the GS model in Korean red pine, training the model with markers of MAF of 0.05 or more, using GBLUP as a predictive model, and including as many environments as possible was effective. The GS of the Korean red pine was as efficient as that of the other tree species. In addition, it was found to have a higher response to selection than traditional PS and FS. Thus, GS is an appropriate alternative to the traditional selection of Korean red pines. The results of this study provide evidence that GS is effective for forest trees even in challenging environments characterized by mountainous terrain, low heritability, and widespread distribution across diverse regions.

## Data availability statement

The original contributions presented in the study are included in the article/**Supplementary Files**. Further inquiries can be directed to the corresponding author.

## Author contributions

HK: Formal analysis, Software, Visualization, Writing – original draft. IK: Conceptualization, Supervision, Writing – review & editing. DS: Conceptualization, Investigation, Methodology, Writing – review & editing. KK: Formal analysis, Writing – review & editing. KC: Data curation, Project administration, Writing – review & editing.

## Supplementary material

Supplementary Figure 2 Distribution of phenotypes by test site and combined data. **(A)** DBH, **(B)** height, **(C)** straightness, and **(D)** volume. Box colors and red X symbols indicate the median and mean of phenotypes respectively in each site. Alphabets in box indicate the Games-Howell *post-hoc* analysis group. T, Taean; C, Chuncheon; G, Gongju; K, Kyeongju; N, Naju; W, Wanju.

Supplementary Figure 3 Heatmaps of coefficient of **(A)** numerator relationship matrix and **(B)** genomic realized relationship matrix ordered by open-pollinated family and **(C)** distribution of GRM coefficients according to their corresponding NRM coefficients. Symbol X indicates the mean of the genomic realized relationship coefficient. GRM was prepared with marker filtering according to the default threshold for marker quality suggested by the SNP calling program, MAF of 0.05, and classifications of high resolution (1,164 SNPs) in this figure.

Supplementary Figure 4 Breeding values estimated using ABLUP by site for four traits. **(A)** DBH, **(B)** height, **(C)** straightness, and **(D)** volume. T, Taean; C, Chuncheon; G, Gongju; K, Kyeongju; N, Naju; W, Wanju.
