# Supplementary figures and images for: Genomic selection for growth characteristics in Korean red pine (Pinus densiflora Seibold & Zucc.)
Source: Front Plant Sci. 2024 Jan 23;15:1285094. doi: 10.3389/fpls.2024.1285094 (PMC10844423; doi:10.3389/fpls.2024.1285094)

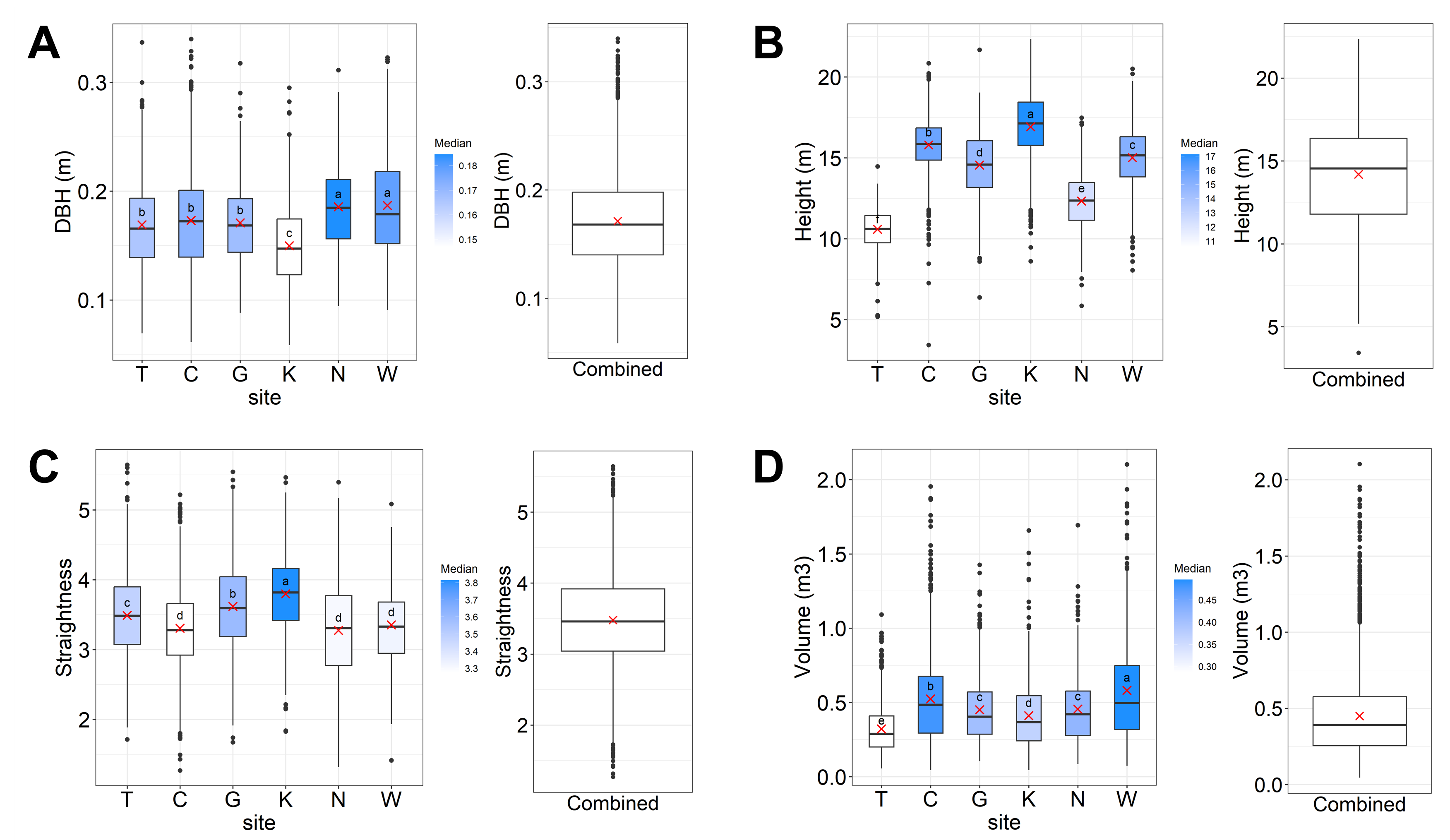

Supplement: Supplementary file 1 [file Image_1.tif]

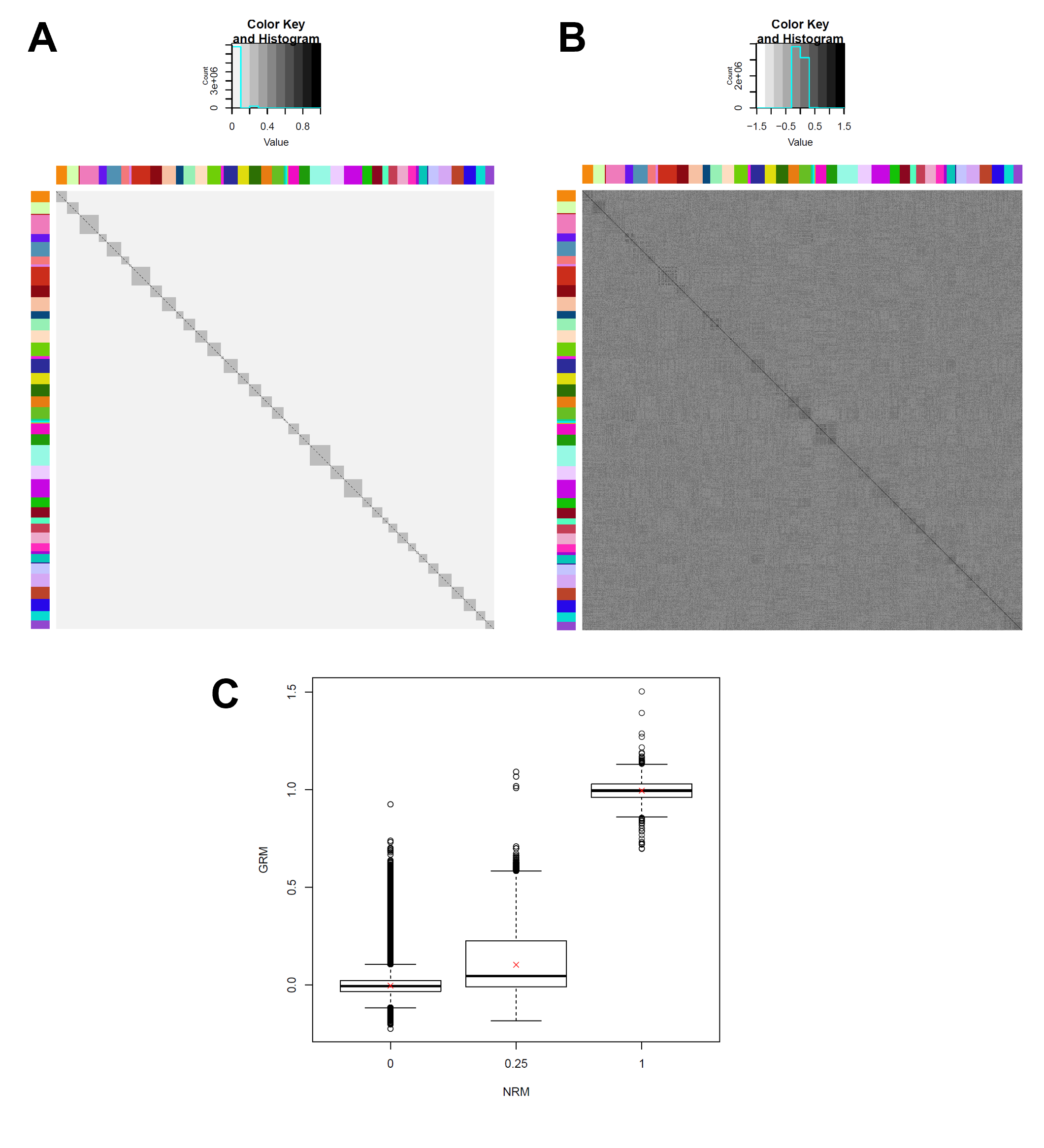

Supplement: Supplementary Figure 2 — Distribution of phenotypes by test site and combined data. (A) DBH, (B) height, (C) straightness, and (D) volume. Box colors and red X symbols indicate the median and mean of phenotypes respectively in each site. Alphabets in box indicate the Games-Howell post-hoc analysis group. T, Taean; C, Chuncheon; G, Gongju; K, Kyeongju; N, Naju; W, Wanju. [file Image_2.tif]

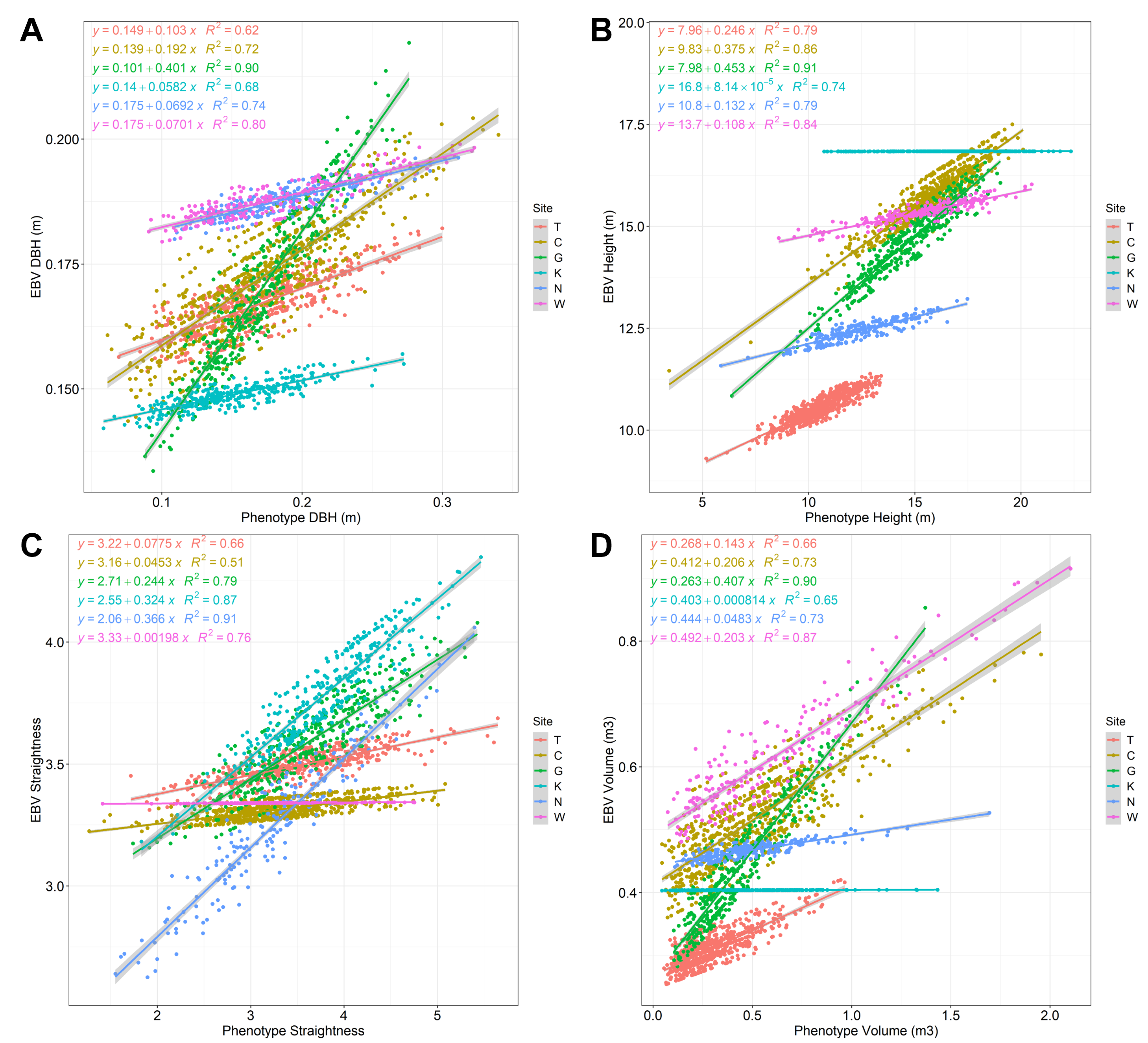

Supplement: Supplementary Figure 3 — Heatmaps of coefficient of (A) numerator relationship matrix and (B) genomic realized relationship matrix ordered by open-pollinated family and (C) distribution of GRM coefficients according to their corresponding NRM coefficients. Symbol X indicates the mean of the genomic realized relationship coefficient. GRM was prepared with marker filtering according to the default threshold for marker quality suggested by the SNP calling program, MAF of 0.05, and classifications of high resolution (1,164 SNPs) in this figure. [file Image_3.tif]

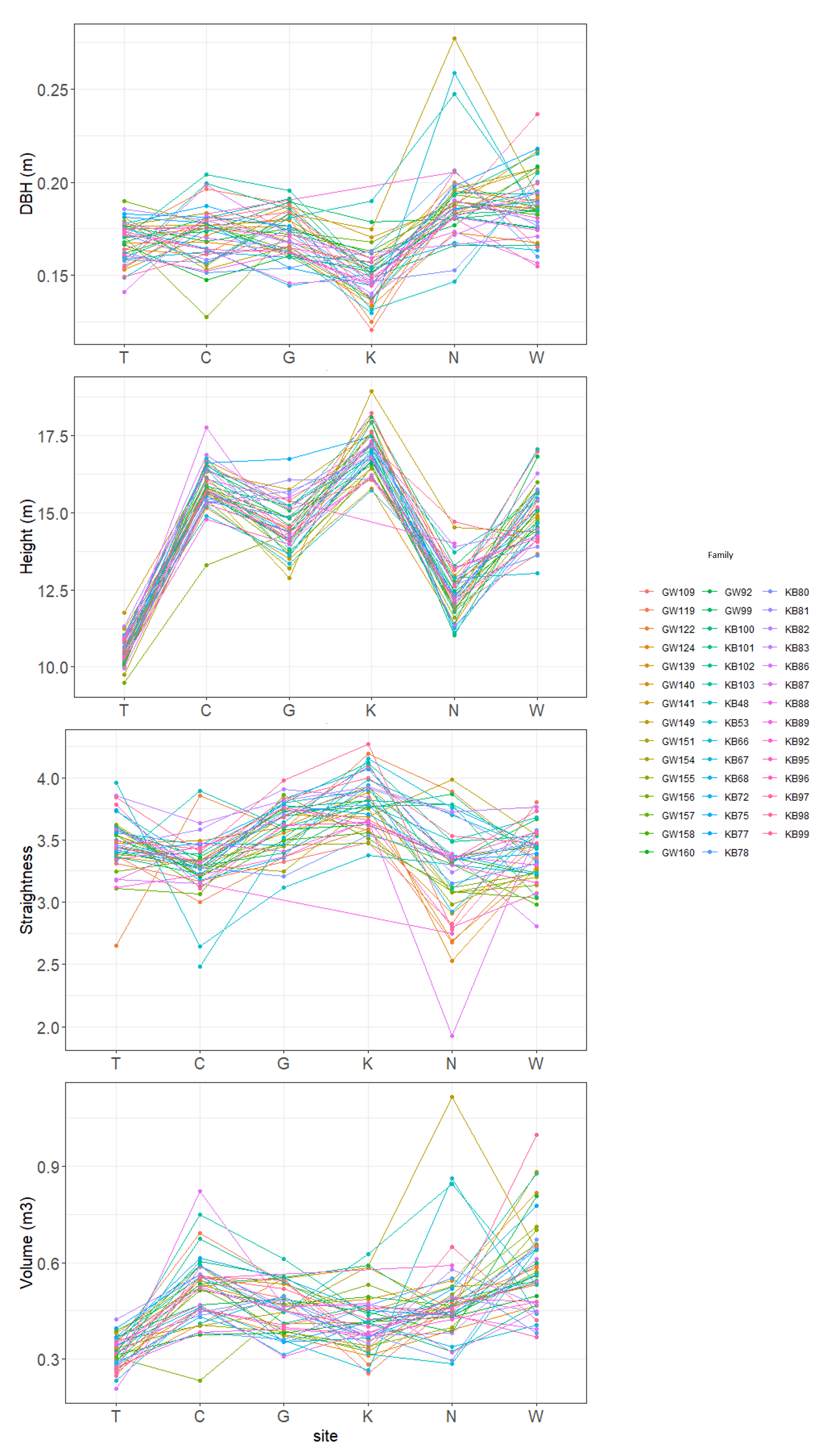

Supplement: Supplementary Figure 4 — Breeding values estimated using ABLUP by site for four traits. (A) DBH, (B) height, (C) straightness, and (D) volume. T, Taean; C, Chuncheon; G, Gongju; K, Kyeongju; N, Naju; W, Wanju. [file Image_4.tif]
