# Supplementary material for: Genomic selection for growth characteristics in Korean red pine (Pinus densiflora Seibold & Zucc.)
Source: Front Plant Sci. 2024 Jan 23;15:1285094. doi: 10.3389/fpls.2024.1285094 (PMC10844423; doi:10.3389/fpls.2024.1285094)
Supplement: Supplementary file 5 [file Table_1.pdf]

Table S1. Open-pollinated families included in the study population

Family [a]	No. of Trees	Family	No. of Trees
GW92	17	KB72	122
GW99	84	KB75	82
GW109	65	KB77	108
GW119	71	KB78	60
GW122	7	KB80	63
GW124	114	KB81	37
GW139	49	KB82	54
GW140	87	KB83	64
GW141	48	KB86	47
GW149	15	KB87	19
GW151	111	KB88	49
GW154	73	KB89	8
GW155	83	KB92	61
GW156	46	KB95	80
GW157	70	KB96	73
GW158	74	KB97	74
GW160	83	KB98	56
KB48	12	KB99	51
KB53	11	KB100	68
KB66	4	KB101	74
KB67	67	KB102	63
KB68	65	KB103	72
Total			
2,643			

[a] GW and KB mean that female parent is plus tree from Gangwon and Kyeongbuk provenance, respectively.

Table S5. Family heritability by ANOVA in each site and combined analysis

Site	DBH	Height	Straightness	Volume
Taean	0.134	0.435	0.259	0.260
Chuncheon	0.304	0.471	0.258	0.276
Gongju	0.342	0.503	0.362	0.408
Kyeongju	0.053	0.089	0.231	0.025
Naju	0.023	0.055	0.429	0.134
Wanju	0.074	0	0.310	0.088
Combined	0.427	0.733	0.639	0.545

Table S6. GBLUP accuracy and predictive ability according to the marker quality threshold

Trait	Site [a]	Accuracy			Predictive ability		
		loose	moderate	strict	loose	moderate	strict
DBH	T	0.29 (0.04)	0.28 (0.04)	0.22 (0.04)	0.11 (0.04)	0.05 (0.05)	0.02 (0.04)
	C	0.5 (0.04)	0.44 (0.03)	0.38 (0.04)	0.29 (0.05)	0.21 (0.04)	0.19 (0.05)
	G	0.33 (0.04)	0.31 (0.03)	0.24 (0.04)	0.18 (0.05)	0.17 (0.04)	0.11 (0.05)
	K	0.25 (0.06)	0.22 (0.08)	0.17 (0.07)	0.05 (0.05)	0.05 (0.08)	0.03 (0.08)
	N	0.44 (0.05)	0.36 (0.04)	0.37 (0.03)	0.32 (0.06)	0.25 (0.03)	0.24 (0.03)
	W	0.25 (0.05)	0.07 (0.05)	0.02 (0.06)	0.19 (0.06)	-0.03 (0.05)	-0.08 (0.06)
Height	T	0.5 (0.04)	0.33 (0.04)	0.3 (0.04)	0.44 (0.04)	0.16 (0.03)	0.15 (0.03)
	C	0.42 (0.01)	0.36 (0.03)	0.33 (0.02)	0.26 (0.03)	0.2 (0.04)	0.18 (0.03)
	G	0.48 (0.04)	0.37 (0.05)	0.35 (0.04)	0.41 (0.05)	0.26 (0.05)	0.26 (0.05)
	K	0.25 (0.06)	0.07 (0.07)	0.03 (0.06)	0.19 (0.07)	-0.02 (0.08)	-0.05 (0.07)
	N	0.44 (0.04)	0.43 (0.03)	0.31 (0.03)	0.32 (0.05)	0.3 (0.04)	0.21 (0.03)
	W	0.16 (0.04)	0.17 (0.05)	0.12 (0.06)	0.06 (0.06)	0.05 (0.04)	0.03 (0.05)
Straight-ness	T	0.32 (0.04)	0.25 (0.04)	0.2 (0.03)	0.1 (0.04)	0.01 (0.05)	0 (0.04)
	C	0.43 (0.03)	0.37 (0.03)	0.31 (0.03)	0.17 (0.03)	0.11 (0.03)	0.1 (0.04)
	G	0.34 (0.03)	0.28 (0.03)	0.22 (0.03)	0.21 (0.04)	0.16 (0.03)	0.11 (0.04)
	K	0.36 (0.05)	0.34 (0.04)	0.34 (0.05)	0.21 (0.05)	0.19 (0.05)	0.2 (0.05)
	N	0.29 (0.05)	0.21 (0.06)	0.16 (0.07)	0.18 (0.05)	0.11 (0.06)	0.06 (0.07)
	W	0.26 (0.06)	0.26 (0.05)	0.28 (0.05)	0.17 (0.05)	0.17 (0.04)	0.19 (0.05)
Volume	T	0.34 (0.05)	0.33 (0.04)	0.29 (0.04)	0.18 (0.05)	0.11 (0.05)	0.09 (0.04)
	C	0.49 (0.03)	0.43 (0.03)	0.37 (0.04)	0.3 (0.04)	0.21 (0.03)	0.19 (0.04)
	G	0.36 (0.03)	0.34 (0.04)	0.29 (0.05)	0.22 (0.05)	0.21 (0.05)	0.17 (0.06)
	K	0.24 (0.07)	0.24 (0.08)	0.17 (0.07)	0.02 (0.06)	0.05 (0.09)	0.02 (0.09)
	N	0.44 (0.05)	0.37 (0.04)	0.36 (0.03)	0.31 (0.05)	0.24 (0.03)	0.19 (0.03)
	W	0.23 (0.05)	0.07 (0.04)	-0.02 (0.05)	0.18 (0.06)	-0.01 (0.03)	-0.09 (0.05)

Mean (standard error) of accuracy and predictive ability from 10-fold cross-validation

[a] T, Taean; C, Chuncheon; G, Gongju; K, Kyeongju; N, Naju; W, Wanju

Table S7. GBLUP accuracy and predictive ability according to the number of randomly selected markers

Trait	Site [a]	No. of markers			
		2K	6K	10K	17K
Accuracy					
DBH	T	0.17 (0.03)	0.27 (0.03)	0.28 (0.03)	0.28 (0.04)
	C	0.35 (0.04)	0.47 (0.03)	0.48 (0.04)	0.5 (0.04)
	G	0.18 (0.05)	0.26 (0.05)	0.28 (0.04)	0.31 (0.04)
	K	0.16 (0.04)	0.23 (0.06)	0.25 (0.06)	0.25 (0.06)
	N	0.38 (0.07)	0.42 (0.06)	0.44 (0.05)	0.44 (0.05)
	W	0.23 (0.05)	0.22 (0.06)	0.25 (0.05)	0.26 (0.05)
Height	T	0.46 (0.03)	0.46 (0.04)	0.49 (0.04)	0.51 (0.03)
	C	0.34 (0.03)	0.38 (0.02)	0.42 (0.01)	0.42 (0.01)
	G	0.37 (0.06)	0.43 (0.04)	0.44 (0.06)	0.46 (0.04)
	K	0.11 (0.07)	0.18 (0.06)	0.24 (0.06)	0.24 (0.06)
	N	0.37 (0.06)	0.4 (0.03)	0.41 (0.05)	0.45 (0.04)
	W	0.09 (0.06)	0.18 (0.05)	0.19 (0.06)	0.22 (0.06)
Straight-ness	T	0.24 (0.04)	0.31 (0.04)	0.32 (0.04)	0.32 (0.04)
	C	0.32 (0.04)	0.37 (0.03)	0.41 (0.03)	0.43 (0.03)
	G	0.24 (0.04)	0.31 (0.03)	0.34 (0.03)	0.35 (0.04)
	K	0.25 (0.04)	0.31 (0.05)	0.32 (0.05)	0.34 (0.04)
	N	0.15 (0.03)	0.21 (0.05)	0.24 (0.05)	0.26 (0.05)
	W	0.23 (0.06)	0.29 (0.06)	0.29 (0.06)	0.26 (0.06)
Volume	T	0.24 (0.04)	0.3 (0.04)	0.32 (0.03)	0.33 (0.04)
	C	0.36 (0.04)	0.45 (0.03)	0.47 (0.03)	0.49 (0.03)
	G	0.23 (0.03)	0.31 (0.04)	0.32 (0.04)	0.35 (0.03)
	K	0.13 (0.05)	0.21 (0.07)	0.24 (0.07)	0.24 (0.07)
	N	0.35 (0.07)	0.43 (0.05)	0.44 (0.05)	0.45 (0.04)
	W	0.21 (0.04)	0.21 (0.05)	0.25 (0.05)	0.26 (0.05)
Predictive ability					
DBH	T	0 (0.03)	0.1 (0.03)	0.07 (0.03)	0.09 (0.03)
	C	0.21 (0.05)	0.28 (0.04)	0.3 (0.04)	0.31 (0.05)
	G	0.1 (0.05)	0.1 (0.07)	0.12 (0.05)	0.16 (0.05)
	K	0 (0.04)	0.05 (0.05)	0.04 (0.05)	0.05 (0.05)
	N	0.33 (0.06)	0.31 (0.06)	0.32 (0.05)	0.32 (0.06)
	W	0.18 (0.07)	0.18 (0.06)	0.19 (0.06)	0.2 (0.06)

Table S7. (Continued)

Trait	Site [a]	No. of markers			
		2K	6K	10K	17K
Height	T	0.43 (0.04)	0.42 (0.04)	0.44 (0.04)	0.46 (0.04)
	C	0.21 (0.02)	0.24 (0.03)	0.27 (0.03)	0.27 (0.03)
	G	0.33 (0.07)	0.35 (0.05)	0.37 (0.06)	0.4 (0.05)
	K	0.08 (0.06)	0.08 (0.07)	0.18 (0.07)	0.16 (0.07)
	N	0.27 (0.06)	0.28 (0.05)	0.28 (0.06)	0.33 (0.05)
	W	0 (0.06)	0.08 (0.06)	0.09 (0.07)	0.11 (0.07)
Straight-ness	T	0.06 (0.04)	0.15 (0.04)	0.11 (0.04)	0.11 (0.04)
	C	0.15 (0.04)	0.13 (0.02)	0.17 (0.03)	0.17 (0.03)
	G	0.14 (0.04)	0.2 (0.04)	0.21 (0.03)	0.21 (0.04)
	K	0.11 (0.05)	0.17 (0.05)	0.17 (0.05)	0.18 (0.05)
	N	0.05 (0.04)	0.11 (0.06)	0.12 (0.05)	0.14 (0.06)
	W	0.15 (0.06)	0.24 (0.05)	0.21 (0.05)	0.17 (0.06)
Volume	T	0.1 (0.04)	0.15 (0.04)	0.15 (0.04)	0.16 (0.05)
	C	0.21 (0.04)	0.28 (0.04)	0.31 (0.04)	0.32 (0.04)
	G	0.15 (0.05)	0.16 (0.06)	0.17 (0.05)	0.21 (0.05)
	K	-0.03 (0.05)	0.01 (0.07)	0.01 (0.07)	0.02 (0.06)
	N	0.27 (0.05)	0.31 (0.05)	0.31 (0.04)	0.32 (0.05)
	W	0.16 (0.06)	0.18 (0.06)	0.19 (0.06)	0.2 (0.05)

Mean (standard error) of accuracy and predictive ability from 10-fold cross-validation

[a] T, Taean; C, Chuncheon; G, Gongju; K, Kyeongju; N, Naju; W, Wanju

Table S8. GBLUP accuracy and predictive ability according to the marker selection based on minor allele frequency

Trait	Site [a]	Minor allele frequency			
		maf≥0.25	0.25>maf ≥0.05	0.05>maf ≥0.0005	0.0005>maf >0
Accuracy					
DBH	T	0.27 (0.04)	0.29 (0.04)	0.28 (0.04)	0.28 (0.04)
	C	0.48 (0.03)	0.5 (0.04)	0.5 (0.04)	0.5 (0.04)
	G	0.3 (0.04)	0.33 (0.04)	0.31 (0.04)	0.31 (0.04)
	K	0.21 (0.07)	0.25 (0.06)	0.25 (0.06)	0.25 (0.06)
	N	0.41 (0.06)	0.44 (0.05)	0.44 (0.05)	0.44 (0.05)
	W	0.25 (0.05)	0.25 (0.05)	0.26 (0.05)	0.26 (0.05)
Height	T	0.43 (0.03)	0.5 (0.04)	0.51 (0.03)	0.51 (0.03)
	C	0.39 (0.02)	0.42 (0.01)	0.42 (0.01)	0.42 (0.01)
	G	0.43 (0.05)	0.48 (0.04)	0.46 (0.04)	0.46 (0.04)
	K	0.14 (0.07)	0.25 (0.06)	0.24 (0.06)	0.24 (0.06)
	N	0.4 (0.04)	0.44 (0.04)	0.45 (0.04)	0.45 (0.04)
	W	0.15 (0.05)	0.16 (0.04)	0.22 (0.06)	0.22 (0.06)
Straight-ness	T	0.31 (0.03)	0.32 (0.04)	0.32 (0.04)	0.32 (0.04)
	C	0.42 (0.03)	0.43 (0.03)	0.43 (0.03)	0.43 (0.03)
	G	0.32 (0.04)	0.34 (0.03)	0.35 (0.04)	0.35 (0.04)
	K	0.32 (0.05)	0.36 (0.05)	0.34 (0.04)	0.34 (0.04)
	N	0.24 (0.06)	0.29 (0.05)	0.26 (0.05)	0.26 (0.05)
	W	0.21 (0.06)	0.26 (0.06)	0.26 (0.06)	0.26 (0.06)
Volume	T	0.31 (0.04)	0.34 (0.05)	0.33 (0.04)	0.33 (0.04)
	C	0.47 (0.03)	0.49 (0.03)	0.49 (0.03)	0.49 (0.03)
	G	0.33 (0.04)	0.36 (0.03)	0.35 (0.03)	0.35 (0.03)
	K	0.22 (0.07)	0.24 (0.07)	0.24 (0.07)	0.24 (0.07)
	N	0.41 (0.05)	0.44 (0.05)	0.45 (0.04)	0.45 (0.04)
	W	0.22 (0.05)	0.23 (0.05)	0.26 (0.05)	0.26 (0.05)
Predictive ability					
DBH	T	0.08 (0.03)	0.11 (0.04)	0.09 (0.03)	0.09 (0.03)
	C	0.27 (0.05)	0.29 (0.05)	0.31 (0.05)	0.31 (0.05)
	G	0.16 (0.05)	0.18 (0.05)	0.16 (0.05)	0.16 (0.05)
	K	-0.01 (0.06)	0.05 (0.05)	0.05 (0.05)	0.05 (0.05)
	N	0.3 (0.06)	0.32 (0.06)	0.32 (0.06)	0.32 (0.06)
	W	0.17 (0.06)	0.19 (0.06)	0.2 (0.06)	0.2 (0.06)

Table S8. (Continued)

Trait	Site [a]	Minor allele frequency			
		maf≥0.25	0.25>maf ≥0.05	0.05>maf ≥0.0005	0.0005>maf >0
Height	T	0.36 (0.04)	0.44 (0.04)	0.46 (0.04)	0.46 (0.04)
	C	0.23 (0.03)	0.26 (0.03)	0.27 (0.03)	0.27 (0.03)
	G	0.34 (0.06)	0.41 (0.05)	0.4 (0.05)	0.4 (0.05)
	K	0 (0.08)	0.19 (0.07)	0.16 (0.07)	0.16 (0.07)
	N	0.25 (0.05)	0.32 (0.05)	0.33 (0.05)	0.33 (0.05)
	W	0.04 (0.06)	0.06 (0.06)	0.11 (0.07)	0.11 (0.07)
Straight-ness	T	0.11 (0.04)	0.1 (0.04)	0.11 (0.04)	0.11 (0.04)
	C	0.15 (0.04)	0.17 (0.03)	0.17 (0.03)	0.17 (0.03)
	G	0.19 (0.04)	0.21 (0.04)	0.21 (0.04)	0.21 (0.04)
	K	0.17 (0.05)	0.21 (0.05)	0.18 (0.05)	0.18 (0.05)
	N	0.12 (0.06)	0.18 (0.05)	0.14 (0.06)	0.14 (0.06)
	W	0.13 (0.05)	0.17 (0.05)	0.17 (0.06)	0.17 (0.06)
Volume	T	0.13 (0.04)	0.18 (0.05)	0.16 (0.05)	0.16 (0.05)
	C	0.28 (0.05)	0.3 (0.04)	0.32 (0.04)	0.32 (0.04)
	G	0.18 (0.06)	0.22 (0.05)	0.21 (0.05)	0.21 (0.05)
	K	-0.02 (0.07)	0.02 (0.06)	0.02 (0.06)	0.02 (0.06)
	N	0.28 (0.05)	0.31 (0.05)	0.32 (0.05)	0.32 (0.05)
	W	0.15 (0.05)	0.18 (0.06)	0.2 (0.05)	0.2 (0.05)

Mean (standard error) of accuracy and predictive ability from 10-fold cross-validation

[a] T, Taean; C, Chuncheon; G, Gongju; K, Kyeongju; N, Naju; W, Wanju

Table S9. Accuracy and predictive ability according to predictive models

Trait	Site [a]	Predictive model [b]						
		ABLUP	GBLUP	BRR	BL	Bayes A	Bayes B	Bayes C
Accuracy								
DBH	T	0.68 (0.02)	0.29 (0.04)	0.28 (0.04)	0.3 (0.04)	0.28 (0.04)	0.28 (0.04)	0.28 (0.04)
	C	0.64 (0.02)	0.5 (0.04)	0.49 (0.04)	0.5 (0.04)	0.49 (0.04)	0.49 (0.04)	0.49 (0.04)
	G	0.51 (0.05)	0.33 (0.04)	0.33 (0.03)	0.32 (0.04)	0.33 (0.03)	0.33 (0.04)	0.33 (0.03)
	K	0.57 (0.05)	0.25 (0.06)	0.24 (0.05)	0.25 (0.06)	0.24 (0.06)	0.23 (0.06)	0.24 (0.06)
	N	0.55 (0.05)	0.44 (0.05)	0.44 (0.05)	0.45 (0.05)	0.44 (0.05)	0.44 (0.06)	0.44 (0.05)
	W	0.49 (0.04)	0.25 (0.05)	0.23 (0.05)	0.24 (0.05)	0.23 (0.04)	0.23 (0.05)	0.24 (0.05)
Height	T	0.59 (0.03)	0.5 (0.04)	0.5 (0.04)	0.5 (0.04)	0.5 (0.04)	0.5 (0.04)	0.5 (0.04)
	C	0.57 (0.03)	0.42 (0.01)	0.41 (0.01)	0.42 (0.01)	0.42 (0.01)	0.42 (0.01)	0.42 (0.01)
	G	0.53 (0.05)	0.48 (0.04)	0.49 (0.04)	0.49 (0.04)	0.49 (0.04)	0.5 (0.04)	0.49 (0.04)
	K	0.32 (0.07)	0.25 (0.06)	0.24 (0.06)	0.24 (0.06)	0.24 (0.06)	0.25 (0.06)	0.24 (0.06)
	N	0.5 (0.03)	0.44 (0.04)	0.45 (0.04)	0.46 (0.04)	0.45 (0.04)	0.46 (0.04)	0.46 (0.04)
	W	0.42 (0.09)	0.16 (0.04)	0.17 (0.06)	0.16 (0.06)	0.16 (0.06)	0.15 (0.06)	0.17 (0.05)
Straight-ness	T	0.62 (0.02)	0.32 (0.04)	0.31 (0.03)	0.31 (0.03)	0.32 (0.03)	0.31 (0.03)	0.31 (0.04)
	C	0.72 (0.02)	0.43 (0.03)	0.41 (0.03)	0.42 (0.03)	0.42 (0.03)	0.4 (0.04)	0.41 (0.03)
	G	0.61 (0.02)	0.34 (0.03)	0.33 (0.04)	0.34 (0.03)	0.33 (0.03)	0.33 (0.03)	0.34 (0.03)
	K	0.56 (0.03)	0.36 (0.05)	0.35 (0.04)	0.36 (0.05)	0.36 (0.05)	0.36 (0.05)	0.35 (0.05)
	N	0.48 (0.04)	0.29 (0.05)	0.29 (0.05)	0.27 (0.05)	0.29 (0.05)	0.3 (0.05)	0.29 (0.05)
	W	0.52 (0.05)	0.26 (0.06)	0.27 (0.06)	0.27 (0.06)	0.26 (0.06)	0.26 (0.06)	0.26 (0.06)

Table S9. (Continued)

Trait	Site	Predictive model *						
		ABLUP	GBLUP	BRR	BL	Bayes A	Bayes B	Bayes C
Volume	T	0.68 (0.01)	0.34 (0.05)	0.33 (0.04)	0.33 (0.04)	0.33 (0.04)	0.32 (0.05)	0.33 (0.05)
	C	0.65 (0.02)	0.49 (0.03)	0.48 (0.03)	0.49 (0.03)	0.48 (0.03)	0.47 (0.03)	0.48 (0.03)
	G	0.52 (0.04)	0.36 (0.03)	0.36 (0.03)	0.35 (0.03)	0.36 (0.03)	0.35 (0.03)	0.36 (0.03)
	K	0.55 (0.05)	0.24 (0.07)	0.24 (0.06)	0.26 (0.06)	0.24 (0.06)	0.24 (0.06)	0.25 (0.06)
	N	0.56 (0.04)	0.44 (0.05)	0.43 (0.05)	0.45 (0.04)	0.44 (0.05)	0.43 (0.05)	0.43 (0.05)
	W	0.44 (0.04)	0.23 (0.05)	0.2 (0.04)	0.22 (0.05)	0.22 (0.04)	0.22 (0.05)	0.21 (0.04)
Predictive ability								
DBH	T	0.11 (0.04)	0.11 (0.04)	0.12 (0.04)	0.11 (0.04)	0.12 (0.04)	0.11 (0.04)	0.11 (0.04)
	C	0.15 (0.03)	0.29 (0.05)	0.28 (0.05)	0.29 (0.05)	0.29 (0.05)	0.28 (0.05)	0.29 (0.05)
	G	0.21 (0.07)	0.18 (0.05)	0.19 (0.05)	0.17 (0.05)	0.19 (0.05)	0.18 (0.05)	0.18 (0.05)
	K	0.04 (0.07)	0.05 (0.05)	0.04 (0.05)	0.05 (0.05)	0.03 (0.05)	0.02 (0.05)	0.04 (0.05)
	N	0.08 (0.06)	0.32 (0.06)	0.33 (0.06)	0.32 (0.06)	0.32 (0.06)	0.32 (0.06)	0.32 (0.06)
	W	0.06 (0.06)	0.19 (0.06)	0.15 (0.05)	0.17 (0.06)	0.15 (0.05)	0.15 (0.05)	0.16 (0.05)
Height	T	0.17 (0.05)	0.44 (0.04)	0.44 (0.04)	0.44 (0.04)	0.44 (0.04)	0.44 (0.04)	0.44 (0.04)
	C	0.22 (0.04)	0.26 (0.03)	0.26 (0.03)	0.27 (0.03)	0.27 (0.03)	0.27 (0.02)	0.26 (0.03)
	G	0.25 (0.06)	0.41 (0.05)	0.41 (0.05)	0.42 (0.05)	0.42 (0.04)	0.43 (0.04)	0.42 (0.05)
	K	-0.18 (0.07)	0.19 (0.07)	0.19 (0.07)	0.18 (0.07)	0.19 (0.07)	0.19 (0.07)	0.19 (0.07)
	N	0.07 (0.03)	0.32 (0.05)	0.32 (0.05)	0.33 (0.05)	0.32 (0.05)	0.33 (0.05)	0.33 (0.05)
	W	0.07 (0.09)	0.06 (0.06)	0.05 (0.07)	0.06 (0.06)	0.06 (0.07)	0.04 (0.07)	0.06 (0.06)

Table S9. (Continued)

Trait	Site	Predictive model *						
		ABLUP	GBLUP	BRR	BL	Bayes A	Bayes B	Bayes C
Straight-ness	T	0.05 (0.04)	0.1 (0.04)	0.09 (0.03)	0.1 (0.04)	0.1 (0.04)	0.09 (0.03)	0.1 (0.04)
	C	0.06 (0.03)	0.17 (0.03)	0.17 (0.03)	0.16 (0.03)	0.17 (0.03)	0.16 (0.03)	0.16 (0.03)
	G	0.2 (0.03)	0.21 (0.04)	0.2 (0.04)	0.21 (0.04)	0.21 (0.04)	0.2 (0.04)	0.2 (0.04)
	K	0.22 (0.05)	0.21 (0.05)	0.21 (0.05)	0.22 (0.05)	0.21 (0.05)	0.21 (0.05)	0.21 (0.05)
	N	0.18 (0.05)	0.18 (0.05)	0.19 (0.05)	0.16 (0.05)	0.2 (0.05)	0.2 (0.05)	0.2 (0.05)
	W	0.04 (0.07)	0.17 (0.05)	0.17 (0.05)	0.17 (0.06)	0.16 (0.05)	0.17 (0.04)	0.17 (0.05)
Volume	T	0.14 (0.04)	0.18 (0.05)	0.18 (0.05)	0.18 (0.05)	0.18 (0.05)	0.17 (0.05)	0.18 (0.05)
	C	0.17 (0.03)	0.3 (0.04)	0.29 (0.04)	0.3 (0.05)	0.29 (0.05)	0.28 (0.04)	0.29 (0.04)
	G	0.22 (0.06)	0.22 (0.05)	0.23 (0.04)	0.21 (0.05)	0.23 (0.05)	0.22 (0.05)	0.22 (0.04)
	K	-0.02 (0.07)	0.02 (0.06)	0.02 (0.07)	0.03 (0.06)	0.01 (0.07)	0.01 (0.06)	0.02 (0.06)
	N	0.06 (0.05)	0.31 (0.05)	0.31 (0.05)	0.31 (0.04)	0.31 (0.05)	0.3 (0.05)	0.3 (0.05)
	W	0.09 (0.06)	0.18 (0.06)	0.12 (0.05)	0.16 (0.05)	0.15 (0.05)	0.16 (0.05)	0.14 (0.05)

Mean (standard error) of accuracy and predictive ability from 10-fold cross-validation

[a] T, Taean; C, Chuncheon; G, Gongju; K, Kyeongju; N, Naju; W, Wanju

[b] ABLUP, additive best linear unbiased prediction; GBLUP, genomic BLUP; BRR, Bayesian ridge regression; BL, Bayesian LASSO

Table S10. GBLUP accuracy and predictive ability by cross-validation fold number.

Trait	Site [a]	Cross-validation folds			
		CV3	CV5	CV10	CV20
Accuracy					
DBH	T	0.3 (0.02)	0.2 (0.04)	0.29 (0.04)	0.3 (0.05)
	C	0.47 (0.04)	0.49 (0.03)	0.5 (0.04)	0.5 (0.03)
	G	0.33 (0.05)	0.35 (0.07)	0.33 (0.04)	0.37 (0.04)
	K	0.18 (0.05)	0.25 (0.07)	0.25 (0.06)	0.27 (0.05)
	N	0.36 (0.06)	0.39 (0.06)	0.44 (0.05)	0.44 (0.06)
	W	0.2 (0.09)	0.19 (0.04)	0.25 (0.05)	0.23 (0.07)
Height	T	0.49 (0.04)	0.51 (0.03)	0.5 (0.04)	0.49 (0.03)
	C	0.41 (0.05)	0.41 (0.04)	0.42 (0.01)	0.43 (0.02)
	G	0.48 (0.01)	0.47 (0.02)	0.48 (0.04)	0.5 (0.03)
	K	0.21 (0.05)	0.19 (0.08)	0.25 (0.06)	0.22 (0.05)
	N	0.36 (0.03)	0.41 (0.03)	0.44 (0.04)	0.41 (0.04)
	W	0.14 (0.07)	0.18 (0.06)	0.16 (0.04)	0.21 (0.06)
Straight-ness	T	0.3 (0.01)	0.29 (0.04)	0.32 (0.04)	0.3 (0.05)
	C	0.39 (0.05)	0.42 (0.03)	0.43 (0.03)	0.42 (0.03)
	G	0.33 (0.04)	0.32 (0.04)	0.34 (0.03)	0.34 (0.03)
	K	0.37 (0.02)	0.34 (0.04)	0.36 (0.05)	0.37 (0.05)
	N	0.22 (0.05)	0.3 (0.03)	0.29 (0.05)	0.31 (0.06)
	W	0.2 (0.07)	0.23 (0.07)	0.26 (0.06)	0.26 (0.06)
Volume	T	0.33 (0.01)	0.27 (0.05)	0.34 (0.05)	0.33 (0.05)
	C	0.47 (0.04)	0.47 (0.02)	0.49 (0.03)	0.49 (0.03)
	G	0.36 (0.04)	0.35 (0.06)	0.36 (0.03)	0.39 (0.04)
	K	0.16 (0.03)	0.24 (0.08)	0.24 (0.07)	0.25 (0.06)
	N	0.35 (0.07)	0.4 (0.04)	0.44 (0.05)	0.46 (0.05)
	W	0.17 (0.1)	0.18 (0.04)	0.23 (0.05)	0.23 (0.07)
Predictive ability					
DBH	T	0.13 (0.03)	0.03 (0.02)	0.11 (0.04)	0.13 (0.04)
	C	0.27 (0.03)	0.27 (0.03)	0.29 (0.05)	0.29 (0.04)
	G	0.18 (0.06)	0.21 (0.07)	0.18 (0.05)	0.21 (0.05)
	K	-0.04 (0.03)	0.04 (0.05)	0.05 (0.05)	0.07 (0.06)
	N	0.25 (0.06)	0.28 (0.04)	0.32 (0.06)	0.32 (0.05)
	W	0.13 (0.12)	0.14 (0.05)	0.19 (0.06)	0.18 (0.06)

Table S10. (Continued)

Trait	Site [a]	Cross-validation folds			
		CV3	CV5	CV10	CV20
Height	T	0.42 (0.02)	0.44 (0.05)	0.44 (0.04)	0.43 (0.03)
	C	0.27 (0.06)	0.26 (0.04)	0.26 (0.03)	0.27 (0.03)
	G	0.39 (0.02)	0.38 (0.02)	0.41 (0.05)	0.43 (0.04)
	K	0.18 (0.03)	0.13 (0.08)	0.19 (0.07)	0.18 (0.06)
	N	0.27 (0.06)	0.29 (0.04)	0.32 (0.05)	0.32 (0.04)
	W	0.06 (0.06)	0.1 (0.06)	0.06 (0.06)	0.11 (0.07)
Straight-ness	T	0.11 (0.02)	0.11 (0.05)	0.1 (0.04)	0.09 (0.04)
	C	0.14 (0.05)	0.16 (0.02)	0.17 (0.03)	0.17 (0.03)
	G	0.19 (0.03)	0.19 (0.03)	0.21 (0.04)	0.2 (0.04)
	K	0.23 (0.04)	0.19 (0.03)	0.21 (0.05)	0.22 (0.05)
	N	0.1 (0.03)	0.18 (0.03)	0.18 (0.05)	0.21 (0.07)
	W	0.15 (0.01)	0.15 (0.07)	0.17 (0.05)	0.17 (0.06)
Volume	T	0.18 (0.04)	0.11 (0.03)	0.18 (0.05)	0.18 (0.05)
	C	0.28 (0.04)	0.27 (0.03)	0.3 (0.04)	0.3 (0.04)
	G	0.23 (0.07)	0.22 (0.06)	0.22 (0.05)	0.24 (0.05)
	K	-0.07 (0)	0.01 (0.05)	0.02 (0.06)	0.02 (0.06)
	N	0.23 (0.07)	0.29 (0.03)	0.31 (0.05)	0.35 (0.05)
	W	0.1 (0.11)	0.13 (0.05)	0.18 (0.06)	0.17 (0.06)

Mean (standard error) of accuracy and predictive ability from 3, 5, 10, 20-fold cross-validation

[a] T, Taean; C, Chuncheon; G, Gongju; K, Kyeongju; N, Naju; W, Wanju

Table S11. GBLUP accuracy and predictive ability according to the environment of training and test population

Trait	Site [a]	Accuracy		Predictive ability	
		within	between	within	between
DBH	T	0.29 (0.04)	0.42 (0.02)	0.11 (0.04)	0.05 (0.03)
	C	0.5 (0.04)	0.45 (0.03)	0.29 (0.05)	0.17 (0.03)
	G	0.33 (0.04)	0.39 (0.05)	0.18 (0.05)	0.09 (0.04)
	K	0.25 (0.06)	0.49 (0.05)	0.05 (0.05)	0.1 (0.07)
	N	0.44 (0.05)	0.41 (0.05)	0.32 (0.06)	0.1 (0.07)
	W	0.25 (0.05)	0.34 (0.06)	0.19 (0.06)	0.08 (0.03)
	Combined	0.38 (0.02)		0.12 (0.02)	
Height	T	0.5 (0.04)	0.46 (0.04)	0.44 (0.04)	0.16 (0.04)
	C	0.42 (0.01)	0.46 (0.04)	0.26 (0.03)	0.19 (0.04)
	G	0.48 (0.04)	0.43 (0.04)	0.41 (0.05)	0.18 (0.04)
	K	0.25 (0.06)	0.44 (0.04)	0.19 (0.07)	0.17 (0.05)
	N	0.44 (0.04)	0.37 (0.05)	0.32 (0.05)	0.11 (0.06)
	W	0.16 (0.04)	0.45 (0.05)	0.06 (0.06)	0.24 (0.06)
	Combined	0.48 (0.02)		0.09 (0.02)	
Straight-ness	T	0.32 (0.04)	0.4 (0.03)	0.1 (0.04)	0.09 (0.06)
	C	0.43 (0.03)	0.38 (0.02)	0.17 (0.03)	0.1 (0.03)
	G	0.34 (0.03)	0.52 (0.04)	0.21 (0.04)	0.23 (0.06)
	K	0.36 (0.05)	0.39 (0.04)	0.21 (0.05)	0.1 (0.06)
	N	0.29 (0.05)	0.36 (0.07)	0.18 (0.05)	0.09 (0.07)
	W	0.26 (0.06)	0.41 (0.05)	0.17 (0.05)	0.13 (0.03)
	Combined	0.45 (0.02)		0.18 (0.03)	
Volume	T	0.34 (0.05)	0.46 (0.02)	0.18 (0.05)	0.1 (0.02)
	C	0.49 (0.03)	0.47 (0.03)	0.3 (0.04)	0.19 (0.03)
	G	0.36 (0.03)	0.42 (0.05)	0.22 (0.05)	0.13 (0.05)
	K	0.24 (0.07)	0.48 (0.05)	0.02 (0.06)	0.12 (0.06)
	N	0.44 (0.05)	0.41 (0.07)	0.31 (0.05)	0.11 (0.05)
	W	0.23 (0.05)	0.34 (0.06)	0.18 (0.06)	0.13 (0.05)
	Combined	0.42 (0.02)		0.07 (0.01)	

Mean (standard error) of accuracy and predictive ability from 10-fold cross-validation

[a] T, Taean; C, Chuncheon; G, Gongju; K, Kyeongju; N, Naju; W, Wanju

Table S12. Annual genetic gain from phenotypic selection, family selection, and genomic selection in each site for four traits.

Trait	Site [a]	Genetic gain [c]		
		ΔG_{PS} (%)	ΔG_{FS} (%)	ΔG_{GS} (%)
DBH	T	0.209	0.0302	0.2842
	C	0.556	0.1192	0.8532
	G	0.354	0.12	0.3904
	K	0.058	0.0175	0.128
	N	0.530	0.0071	0.6329
	W	0.228	0.0355	0.292
Height	T	0.407	0.0617	0.4767
	C	0.202	0.0738	0.2718
	G	0.450	0.1224	0.5141
	K	0.351	0.0203	0.2328
	N	0.450	0.011	0.4979
	W	0.072	0	0.0726
Straight-ness	T	0.122	0.0632	0.204
	C	0.192	0.0581	0.3434
	G	0.297	0.0842	0.3392
	K	0.298	0.0676	0.3375
	N	0.040	0.0154	0.0371
	W	0.217	0.0709	0.2154
Volume	T	0.677	0.1329	0.8712
	C	1.319	0.209	1.8799
	G	0.909	0.3497	1.0529
	K	0.012	0.0195	0.0837
	N	1.200	0.0998	1.4631
	W	0.528	0.1089	0.587

[a] T, Taean; C, Chuncheon; G, Gongju; K, Kyeongju; N, Naju; W, Wanju

[b] GSAC, GS accuracy, r(GEBV, EBV) EBV was estimated by GBLUP with all phenotype data

[c] Ratio of genetic gain per year to mean. ΔG_{PS}, the genetic gain of phenotypic selection; ΔG_{FS}, the genetic gain of family selection; ΔG_{GS}, the genetic gain of genomic selection.

Table S13. Type-B genetic correlation between the open-pollinated progeny test sites

DBH						
	Taean	Chuncheon	Gongju	Kyeongju	Naju	Wanju
Taean	-					
Chuncheon	0.123	-				
Gongju	-0.071	0.472**	-			
Kyeongju	-0.183	0.062	0.241	-		
Naju	0.062	0.088	-0.030	0.198	-	
Wanju	-0.057	0.197	-0.020	0.041	0.031	-
Height						
	Taean	Chuncheon	Gongju	Kyeongju	Naju	Wanju
Taean	-					
Chuncheon	0.307*	-				
Gongju	0.283	0.188	-			
Kyeongju	-0.078	0.211	0.203	-		
Naju	0.296	0.433**	0.335*	0.306	-	
Wanju	0.050	0.322*	0.061	0.114	-0.007	-
Straightness						
	Taean	Chuncheon	Gongju	Kyeongju	Naju	Wanju
Taean	-					
Chuncheon	0.207	-				
Gongju	0.233	0.351*	-			
Kyeongju	0.210	0.136	0.158	-		
Naju	0.190	0.157	0.074	0.076	-	
Wanju	0.250	0.387**	0.278	-0.126	-0.005	-
Volume						
	Taean	Chuncheon	Gongju	Kyeongju	Naju	Wanju
Taean	-					
Chuncheon	0.181	-				
Gongju	-0.042	0.515***	-			
Kyeongju	-0.207	0.185	0.331*	-		
Naju	0.171	0.191	0.090	0.205	-	
Wanju	-0.023	0.278	0.044	0.131	0.017	-

* 0.01<p-value<0.05, ** 0.001<p-value<0.01, *** p-value<0.001
